# FuChi is a cell cycle biosensor for tracking cell cycle dynamics during avian development

**DOI:** 10.1371/journal.pbio.3004036

**Published:** 2026-10-09

**Authors:** Zoe R. Sudderick, Tiernan Briggs, Shirooza Mubarak, Melinda Van Kerckvoorde, Ana R. Hernandez Rodriguez, Sudeepta K. Panda, Jon Riddell, Cameron Batho-Samblas, Lorna Taylor, Lynn McTeir, Dominique Meunier, Amy Findlay, Flossie S. Roberts, Anna Raper, Asako Sakaue-Sawano, Atsushi Miyawaki, Joe Rainger, Jeffrey J. Schoenebeck, Cornelis J. Weijer, Mike J. McGrew, Denis J. Headon, Megan G. Davey, Richard L. Mort, James D. Glover

**Affiliations:** 1 The Roslin Institute & R(D)SVS, University of Edinburgh, Edinburgh, United Kingdom; 2 Roslin Institute Chicken Embryology (RICE) Group, The Roslin Institute & R(D)SVS, University of Edinburgh, Edinburgh, United Kingdom; 3 Division of Biomedical and Life Sciences, Faculty of Health and Medicine, Lancaster University, Lancaster, United Kingdom; 4 Faculty of Life Sciences, University of Dundee, Dundee, United Kingdom; 5 Gurdon Institute, University of Cambridge, Cambridge, United Kingdom; 6 Division of Physiology and Climatology, ICAR-Indian Veterinary Research Institute, Izatnagar, Uttar Pradesh, India; 7 Laboratory for Cell Function Dynamics, RIKEN Center for Brain Science, Wako, Saitama, Japan; The Institute of Cancer Research, UNITED KINGDOM OF GREAT BRITAIN AND NORTHERN IRELAND

## Abstract

The ability to monitor the proliferative status of live cells both in vitro and in vivo over time has revolutionised our understanding of development, growth and disease. This was first made possible by fluorescent ubiquitination-based cell cycle indicator (Fucci) technology, which distinguishes specific cell cycle phases through the reciprocal degradation of fluorescently tagged, truncated forms of human CDT1 and GMNN proteins. Fucci genetic systems have been successfully implemented in transgenic mice, zebrafish, and axolotls. To date, no viable, stably expressing Fucci line has been developed in an avian species. Although a range of continuously improving Fucci constructs have been developed in recent years, existing in vivo Fucci models remain limited because they rely on older reporter technology that fails to distinguish cells in S, G2, and M phases or to label cells in early G1. As a result, these models can be challenging to interpret and their utility for continuous cell tracking and precise analysis of cell-cycle dynamics is limited. Here, we introduce FuChi, a multicistronic Fucci-expressing chicken line incorporating a newly optimised reporter construct composed of an mCerulean-tagged histone H1.0 linker protein fused via a self-cleaving 2A peptide to the tandem Fucci(CA)2 cell cycle biosensor, with additional epitope tags included for detection in fixed tissues. We show that this system accurately discriminates and permits tracking of cells in G1, S, G2, and M phases both in vitro and in vivo, enabling faithful visualisation of cell cycle status in intact tissues and organs. Using FuChi embryos, we mapped proliferation dynamics across developing tissues, analysed cell cycle states of migrating cells, and performed live imaging of early embryos. These latter experiments revealed that transition from S phase may be a key morphogenetic event during gastrulation as mesendoderm cells egress from the primitive streak to form embryonic structures including the prechordal plate. Pairing this advanced reporter with the intrinsic experimental advantages of the chicken embryo positions FuChi as a premier in vivo system for studying cell-cycle kinetics in development, delivering clear technological improvements over current Fucci models. FuChi chickens provide a powerful new resource for studying embryonic development, organ growth, tissue homeostasis, disease processes, and infection responses.

## Abstract 2

Fucci genetic systems are commonly used to monitor cell proliferation in living cells, but no stably expressing Fucci line has been developed in an avian species. This study develops FuChi, the first avian Fucci reporter line that enables precise cell cycle tracking during embryonic development.

## Introduction

The ability to monitor the proliferative status of cells has transformed our view of development, growth, and disease. Cell cycle control diversifies as development proceeds [1] and is tightly coordinated within tissues, underpinning key aspects of morphogenesis and organogenesis such as lung epithelial branching, ureteric bud development, and digit formation [2–4]. In recent years, live imaging of cell cycle progression has become the gold standard for cell cycle analysis.

The E3 ubiquitin ligases APC^Cdh1^, SCF^Skp2^, and CUL4^Ddb1^ regulate the cell cycle by targeting specific proteins for ubiquitin-mediated degradation. SCF^Skp2^ acts not only as a substrate of APC^Cdh1^ but also directly inhibits it, resulting in reciprocal oscillations in their activities and the levels of their target proteins. APC^Cdh1^ is predominantly active in late mitosis and G1 phase, SCF^Skp2^ functions during S and G2 phases, and CUL4^Ddb1^ is predominantly active during S phase [5–8]. Chromatin licensing and DNA replication factor 1 (CDT1) and Geminin (GMNN), which regulate DNA replication licensing, are targets of SCF^Skp2^/CUL4^Ddb1^ and APC^Cdh1^, respectively, and therefore also exhibit oscillations in their protein levels (Fig 1A and 1B) [9,10]. These dynamics have been exploited to develop the Fucci (Fluorescent Ubiquitination-based Cell Cycle Indicator) systems (Fig 1C and 1D) [11–13].

**Fig 1:**
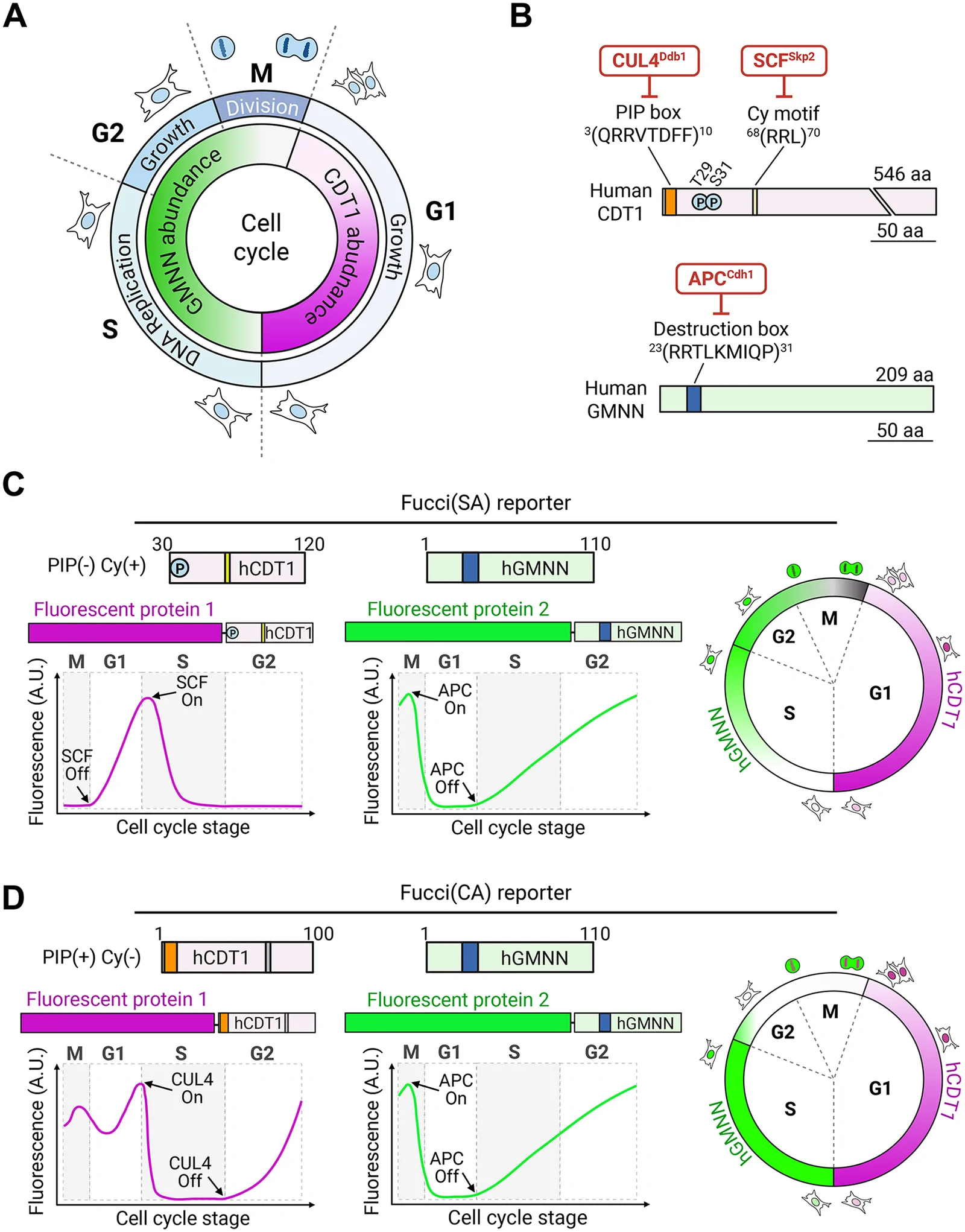
Basis and design of different Fucci reporters. **A)** Schematic of cell cycle stages including the abundance of GMNN and CDT1 proteins, two essential and interacting DNA replication licensing factors that accumulate at distinct phases of the cell cycle. For visualisation purposes, we have chosen to represent the red fluorescence as magenta in all our Fucci reporter system schematics. **B)** Structure of human CDT1 (hCDT1) and human GMNN (hGMNN) proteins, including key features regulating their proteolysis during the cell cycle. The N-terminus of CDT1 is targeted for degradation by two different E3 ubiquitin ligases, CUL4^Ddb1^ and SCF^Skp2^, which bind to the PIP box and Cy motif, respectively. Phosphorylation of key amino acids (Thr29 and Ser31) facilitates binding of SCF^Skp2^. Ubiquitination and proteolysis of GMNN is regulated through the E3 ligase APC^Cdh1^ binding to the destruction box. **C)** Fucci biosensors utilise fusion proteins of hCDT1 and hGMNN, containing truncated forms of the proteins fused with fluorescent markers. In Fucci(SA) systems, an SCF^Skp2^-sensitive hCDT1 probe linked to a fluorescent protein (e.g., mKO2/mCherry) is used in combination with a truncated APC^Cdh1^-sensitive hGMNN fused to a spectrally distinct fluorescent protein (e.g. mAG/mVenus). The expected fluorescent profiles throughout the cell cycle reflect hCDT1 and hGMNN degradation by their respective E3 ligases. Note that double-labelled cells are white and negative cells are grey in the combined schematic. **D)** Fucci(CA) systems combine a CUL4^Ddb1^-sensitive hCDT1 probe and an APC^Cdh1^-sensitive hGMNN probe, which create a distinct fluorescent profile from Fucci(SA) based on the degradation of hCDT1. The grey Cy motif denotes the RRL to AAA change in this probe, which renders the Cy motif nonfunctional. As above, double-labelled cells are white and negative cells are grey in the combined schematic. Schematics created in BioRender. Glover, J. (2026) https://BioRender.com/37cu02a.

The original Fucci(SA) probe pair (Fig 1C) included a fusion of monomeric Kusabira Orange (mKO2) with a truncated human CDT1 (amino acids 30–120), and a fusion of monomeric Azami Green (mAG) with the N-terminal amino acids (1–110) of human GMNN. The mKO2-hCDT1(30/120) probe accumulates in G1 phase, labelling cell nuclei red, and is degraded at the G1–S transition, while the mAG-hGMNN(1/110) probe accumulates during S, G2, and M phases, labelling cell nuclei green, and is rapidly degraded before cytokinesis [13] (Fig 1C).

An improved version of Fucci(SA), sometimes referred to as Fucci2 or Fucci(SA)2, replaces the original mKO2 and mAG fluorescent proteins with mCherry and mVenus, respectively, increasing signal separation and compatibility with other reporters [14]. This probe set was initially expressed from separate constructs, but incorporation of a 2A self-cleaving peptide enabled equimolar expression of reporter proteins from a single promoter (termed Fucci2a or tandem-Fucci(SA)2) and facilitated the development of Cre-inducible mouse models [12,15].

The bicistronic tandem-Fucci(CA) probe pair [11] (Fig 1D) updates the CDT1 probe from Fucci(SA) by removing the SCF^Skp2^-targeted Cy motif and instead including an N-terminal PIP box, which is targeted for degradation in S-phase via CUL4^Ddb1^. This leads to accumulation of the CDT1 probe starting at the S-G2 transition and continuing through G2, M and G1 phases with rapid degradation at the G1/S transition. This results in labelling of G1-phase nuclei as magenta, S-phase nuclei as green and G2 and M-phase nuclei as white with just two sensors [11] (Fig 1D).

Fucci(SA) systems have been used in mouse and human cell lines and mouse models [13,16,17], including tandem-Fucci(SA)2 variants [12,15]. Fucci(SA)-based reporters have also been adapted for zebrafish (zFucci) [18] and axolotls [19] using species-specific CDT1 and GMNN homologues. However, none of these in vivo models label all four cell cycle phases, and detecting the fluorescent proteins in fixed tissues remains challenging. Fucci4 solves the first of these problems by combining four sensors: hCDT1(30–120) degraded by SCF^Skp2^ marks G1, a PIP degron targeted by CUL4^Ddb1^ labels S phase, GMNN(1–110), degraded by APC^Cdh1^, marks S and G2, and an H1.0 linker histone fusion highlights chromatin during mitosis. However, although powerful for in vitro imaging, Fucci4 is not easily deployable in animal models as the sensors are expressed separately [20].

Chickens are a valuable research model because their embryos develop externally, allowing real-time imaging and manipulation, and their early amniote morphology closely parallels that of humans [21,22]. Fucci systems have previously been introduced into cultured chicken fibroblasts and primordial germ cells (PGCs), respectively [23,24], suggesting compatibility with avian species; however, no tractable in vivo germline model system has yet been developed.

Here, we describe a new Fucci-based biosensor, H1.0-Fucci(CA)2, designed to label all four cell cycle phases and incorporating epitope tags for detection in fixed tissues. Using the piggyBac system, we integrated H1.0-Fucci(CA)2 into chicken fibroblasts and PGCs, and demonstrated that it outperforms Fucci(SA)2 by faithfully discriminating between cells in G1, S, G2, and M phases. We further generated a stable line of H1.0-Fucci(CA)2-expressing transgenic chickens (FuChi), enabling, for the first time, in vivo studies of cell cycle progression during embryonic development in an avian model.

## Materials and methods

### H1.0-Fucci(CA)2 construction

To generate pcDNA5-CAG-H1.0-Cerulean-Fucci(CA), a gene fragment encoding the mCerulean fluorescent protein fused to an HA epitope tag and a porcine teschovirus-1 2A peptide (P2A), flanked by 5′ MluI and 3′ HindIII restriction sites, was synthesised by GeneArt (Thermo Fisher). The mCerulean fragment was cloned into pZERO-1 (Thermo Fisher), and the coding sequence of the mouse H1f0 gene (encoding the H1.0 linker histone) was PCR-amplified from mouse cDNA (Table 1) and cloned into the MluI site.

**Table 1:** Oligonucleotide sequences used for cloning and PCR.

Name	Oligonucleotide sequence 5′ - 3′
Oligonucleotide sequences used for cloning*:	
H1.0_For	CGGCAC**ACGCGT**ACCGAGAACTCCACCTCCGCCCCGGC
H1.0_Rev	GCCCTG**GCGCGC**CTTCTTCTTGCTGGCCCTCTTGGCACTCGACTTGGC
mVenus_For	CGGCAC**ATGCAT**GAACAAAAACTCATCTCAGAAGAGGATCTGATGGTGAGCAAGGGCGAGGAGCTGTTCACCGG
hGem_Rev	GACGTC**ACCGGT**TTACAGCGCCTTTCTCCGTTTTTCTGCCACTTCCTTCCAATACTGAGAGG
Polylinker Fw	**ACGCGT**TCGCGA**ACCGGT**TTCGAAGTTTAAAC
Polylinker Rv	GTTTAAACTTCGAAACCGGTTCGCGAACGCGT
Oligonucleotide sequences used for genotyping:	
Fucci internal Fw	GCAATTGGCCGCCACCATGAC
Fucci internal Rv	CTTGCTCACAGCGTAATCTGGAACATCGTATGG
Chromosome 1 Fw	CATAGTTAAATCAAACTCCCACAGG
Chromosome 1 Rv	TGTGCCAAAGTTGTTTCTGACTG
Chromosome 2 Fw	CAGACCGATAAAACACATGCGT
Chromosome 2 Rv	GAGCCACCCAGTTCTGAACA

*Restriction endonuclease sites used for cloning are indicated in bold and underlined.

Subsequently, an mCherry-hCDT1(1/100)PIP(+)Cy(−) gene fragment [11], including a 5′ 6× His tag and HindIII, and 3′ NsiI sites, was synthesised by GeneArt. An mVenus-hGMNN(1/110) fragment [11] was PCR-amplified to incorporate a 5′ Myc tag, NsiI, and 3′ AgeI sites (Table 1). These three components were sequentially cloned into a pcDNA5-Flp backbone modified to contain the CAG promoter in place of CMV [15]. The fidelity of all clones was checked by sequencing.

To generate pB-H1.0-Cerulean-Fucci(CA)2, a polylinker sequence (Table 1) was cloned into pB-H2B-Cerulean-Fucci2a [25] using MluI/PmeI, and the H1.0-Fucci(CA) construct was transferred as an MluI/AgeI fragment.

### Transfection into PGCs

To generate H1.0-Fucci(CA)2 PGCs, 2 µg of pB-H1.0-Cerulean-Fucci(CA)2 was co-transfected with 1 µg of piggyBac Hybase vector [26] into a previously derived line of male PGCs (FR2M, Hy-Line birds) using Lipofectamine 2000 (Life Technologies), and grown in FAOT medium [27]. Stably transduced cells were then selected with puromycin (Sigma-Aldrich) and expanded prior to PGC injection.

### Generation of FuChi embryos

H1.0-Fucci(CA)2 PGCs were micro-injected into Hamburger–Hamilton [28] (HH) stage 15–16 surrogate iCaspase9 sterile hosts as described previously [29]. Briefly, 1 µl of 25 mM B/B compound (Takara Bio) was added to a 50 µl cell suspension (5,000 PGCs/µl) before 1 µl of this suspension was injected into the dorsal aorta of host embryos within windowed eggs. Eggshells were sealed with medical Leukosilk tape (BSN Medical) and incubated until hatch. Founder F_0_ FuChi males were mated with wild-type Hy-Line females to produce heterozygous F_1_ FuChi eggs and offspring. Additionally, F_2_ embryos were generated from subsequent matings of F_1_ adult FuChi males with wild-type Hy-Line females. Both F_1_ and F_2_ embryos were used for downstream analyses. Chicken experiments were conducted under UK Home Office licence PP9565661 and approved by the Roslin Institute Animal Welfare and Ethical Review Board Committee (study numbers #P305 and #P345). The fertility, viability (of fertile eggs), and transgene transmission rates (determined by fluorescence) were recorded.

### Eggs

The FuChi line is maintained by the National Avian Research Facility (NARF), The Roslin Institute, UK. Eggs were incubated vertically in rocking or non-rocking incubators (Masalles) at 37.8 °C. All post-incubation day 14 embryos were treated in accordance with the Animals (Scientific Procedures) Act 1986 schedule 1 culling methods. Fluorescent, and therefore transgenic, embryos were identified using a Nightsea SFA-RB fluorescence adapter and/or a Zeiss Axio Zoom.V16 stereo zoom microscope.

### Cell culture and transfections

DF1 cells were maintained in DMEM (Life Technologies) containing 10% foetal bovine serum (FBS), 2% chicken serum (CS), 1% penicillin/streptomycin. Cells were transfected with either pB-H1.0-Cerulean-Fucci(CA)2 or pB-H2B-Cerulean-Fucci2A [25], together with a vector expressing piggyBac Hybase using Lipofectamine LTX (Life Technologies) as per the manufacturer’s instructions. The cell lines were termed H1.0-Fucci(CA)2-DF1 and H2B-Fucci(SA)2-DF1 in line with Sakaue-Sawano and colleagues [11].

FuChi primary fibroblasts were derived from HH19 limb bud micromasses. Briefly, HH19 embryos were dissected from the egg into DMEM and all limb buds removed. Limb buds were placed in 10× Trypsin (Life Technologies) on ice for 30–40 min, followed by two washes with PBS and removal of the ectoderm with sharpened tungsten needles. Limb bud mesoderm was collected in 100 μl of micromass culture medium (50:50 DMEM/Ham’s F12, 10% FBS, 1% Antibiotic/Antimycotic, 1% L-Glutamine) and dissociated by trituration. 900 μl of cell culture medium was added and the samples were centrifuged at 300 rcf for 2 min. The cell pellet was resuspended in 10 μl of cell culture medium per limb bud. Ten μl drops were added to individual wells of a 6-well glass-bottom plate and incubated for 30 min to 1 hour at 37 °C. Wells were then flooded with micromass culture medium.

To derive FuChi PGCs, 1–2 µl of blood was extracted from HH16 F_1_ FuChi embryos, transferred into FAOT medium and cells expanded as previously described [27].

### Cell treatments

Prior to flow cytometry analysis, H2B-Fucci(SA)2-DF1, H1.0-Fucci(CA)2-DF1 cells or FuChi PGCs were grown in their regular medium conditions as described above. Cells were then treated for 16 hours under control conditions (regular medium plus DMSO), basal conditions (regular culture medium without FBS and CS for DF1, or FAOT medium without growth factors for PGCs), or nocodazole-treated conditions (regular culture medium containing 100 ng/ml nocodazole; Sigma-Aldrich). Following treatment, adherent DF1 cells were dissociated from the flasks, while PGCs were collected. Cells were centrifuged, put through a 70 µm cell strainer, and stained with DAPI (Sigma-Aldrich) for 30 min. Cells were analysed for DNA content and mCherry/mVenus fluorescence using a BD Fortessa ×20.

### Live cell imaging

Primary FuChi limb micromass cultures were grown on glass-bottom culture plates in micromass culture medium and imaged for 72 hours. H2B-Fucci(SA)2-DF1 and H1.0-Fucci(CA)2-DF1 cultures were grown on glass-bottom plates in cell culture medium overnight then imaged for 48 hours. All live imaging was carried out on a Zeiss LSM880 confocal microscope, with z-stack images taken at 10-min intervals. Cell tracking and generation of individual cell montages was carried out using ImageJ/Fiji [30] and the Fucci Tools plugin [31].

### Light sheet imaging and analysis

FuChi eggs were obtained from the NARF and stored at 16 °C until the start of the experiments. The eggs were incubated for 1 hour to equilibrate to 37 °C, after which embryos were isolated on filter paper and mounted in a specially constructed imaging chamber and imaged on a dedicated light sheet microscope as described previously [32]. To image the embryos at 10× magnification, two slightly overlapping scans were performed. During the scanning, the surface of the embryo was kept in focus using a dedicated surface tracking algorithm as previously described [32]. Each scan consisted of 2,500–3,000 successive slices taken at 1.84 µm intervals and imaged at 488 and 562 nm excitation. This procedure allowed us to collect whole embryo images at two wavelengths at 8–10 min intervals.

To process the raw image data, the images were transformed into a rectangular coordinate system and the surface was detected using a surface-finding algorithm. The top 30 z slices were projected onto a plane and the nuclei detected in each channel using a custom MATLAB Laplacian of Gaussian (LoG) blob detector algorithm. Nuclei were classified into three classes, those detected exclusively in either the green or red channel and those detected in both, as determined by their co-localisation. For analysis of variation of mVenus-hGMNN (488 channel) and mCherry-hCDT1 (562 channel) fluorescence during the cell cycle in vivo, selected nuclei were traced manually and their intensities in both channels measured. Individual traces were aligned using the sudden breakdown of mVenus-hGMNN and mCherry-hCDT1 at mitosis. The boundary between the embryonic and extraembryonic area at the start of the experiment was derived from the Lagrangian repeller [33,34] and subsequently evolved for all successive timepoints using calculated tissue velocity [32].

### Whole genome sequencing

Sequencing was carried out as in Oh and colleagues [35]. Briefly, embryonic day 9 livers were snap-frozen on dry ice and stored at −80 °C. Genomic DNA was extracted from the frozen samples using a Monarch HMW DNA Extraction Kit (New England Biolabs) according to the manufacturer’s instructions. Diluted genomic DNA quality was assessed using an Agilent TapeStation, and concentrations were quantified with a Qubit fluorometer. gDNA from 5 samples was pooled. Library preparation and sequencing was carried out by Edinburgh Genomics. The SMRTbell library was sequenced on a Pacific Biosystems Revio platform (25M ZMW, HiFi mode) using a single SMRT cell. A total of 58 Gb of DNA sequence was generated, with an average fragment size of 19 kb and a mean Phred quality score of Q30. Data were returned in BAM and FASTQ file formats (ENA accession: PRJEB98847). To detect the genomic location(s) of transgene insertion, HiFi reads in fastq.gz format were first aligned against the corresponding transgene sequence using Minimap2 (v2.24-r1122) [36]. In total, 69 of 2,983,364 HiFi reads were mapped. Transgene-mapped reads were retained by Samtools (v1.18 using htslib v1.18) [37]. The output file was converted from BAM to FASTQ and the transgene-mapped reads were realigned using Minimap2 against the paternal haplotype of GRCg7w (white leghorn layer, GCF_016700215.2) [38].

### PCR

Insertion sites in chromosomes 1 and 2 revealed by long-read sequencing were confirmed by PCR (see Table 1 for primers) using Phusion polymerase (NEB), and the supplied HF or GC buffers. Detection of transgene insertion on chromosome 1 was carried out with HF buffer with the following conditions: 98 °C/30 s, (98 °C/10 s, 60°C/30 s, 72 °C/30 s) for 33 cycles, 72 °C/5 min. Detection of transgene insertion on chromosome 2 was carried out with GC-rich buffer with the following conditions: 98 °C/30 s, (98 °C/10 s, 64 °C/30 s, 72 °C/30 s) for 30 cycles, 72 °C/5 min. PCR samples were run on 2% agarose gels for assessment.

### Protein sequence comparison

CDT1 and GMNN protein sequences from various species were downloaded from NCBI and aligned in MegAlign Pro 17 (DNAstar) using the MAFFT algorithm.

### Cryo-embedding and paraffin processing

For cryo-processing, FuChi embryos were dissected from the egg, briefly washed in PBS, and fixed with 4% PFA or 10% NBF. Following fixation, embryos were washed in PBS then equilibrated in 15% sucrose/PBS at room temperature, then incubated in 15% sucrose/7.5% gelatin/PBS at 37 °C until fully equilibrated. Blocks of samples embedded in sucrose/gelatin blocks were flash-frozen in isopentane on dry ice, and stored at −80°C. Ten μm cryosections were cut on a Leica CM1950 Cryostat. For imaging, cryosections were incubated in PBS at 37 °C for 15 min to remove gelatin, before mounting in Prolong Gold (Life Technologies) and imaged on a Zeiss LSM880 confocal microscope.

For paraffin embedding, fixed samples (10% NBF or 4% PFA) were washed in PBS, dehydrated through 70%, 90%, and 100% ethanol before clearing in 1:1 ethanol: chloroform, and a final wash in 100% chloroform. Samples were incubated overnight in paraffin wax at 65 °C prior to embedding. 6 μm sections for immunofluorescent analysis were cut using a Thermo Scientific Microm HM 325 microtome.

### Immunofluorescence and fluorescent RNA in situ hybridisation

Immunofluorescent detection of the epitope tags in the H1.0-Fucci(CA)2 biosensor was carried out on formalin-fixed paraffin-embedded (FFPE) sections of FuChi embryos using anti-6× His Tag (Mouse (1:200), Invitrogen #MA1-21315) and anti-Myc Tag (Rabbit (1:100), Cell Signaling Technology #2278) antibodies, or anti-Myc Tag (#2, Mouse (1:500), Cell Signaling Technology #2276). For FFPE sections, after dewaxing, an antigen retrieval step was performed either in EDTA buffer (1 mM EDTA, pH 8.0), Tris-EDTA buffer (10 mM Tris/1 mM EDTA, pH 9.0) or citrate buffer (10 mM sodium citrate, pH 6) using an Antigen Retriever 2100 (Aptum Biologics), or incubation with 5 µg/ml Proteinase K (Invitrogen AM2546) for 20 min at 37 °C. Of note, we tested two different primary antibodies for each epitope tag, across different antigen retrieval conditions. Our results are summarised in Table 2 below. For immunofluorescence on cryosections, samples were equilibrated to room temperature and then incubated for 30 min in PBS at 37 °C to remove gelatin. After these steps, paraffin and cryosections were treated similarly. Sections were permeabilised with TBS containing 0.1%–0.3% Triton X-100 for 10 min, briefly washed in TBS containing 0.1% Tween 20 (TBST), and blocked in 10% goat serum/TBST for one hour at room temperature. Sections were then incubated overnight at 4 °C in primary antibodies (anti-6× His Tag, anti-Myc Tag, or anti-YAP1 (Rabbit (1:200), Proteintech #13584)) diluted in blocking buffer. Samples were washed in TBST before incubation in fluorescent secondary antibodies (Goat anti-rabbit IgG Alexa Fluor 647 (Life Technologies # A-21245), Goat anti-rabbit IgG Alexa Fluor 546 (Life Technologies #A-11035), Goat anti-rabbit IgG Alexa Fluor 488 (Life Technologies #A-11034), Goat anti-mouse IgG Alexa Fluor 647 (Life Technologies #A-21236), or Goat anti-mouse IgG Alexa Fluor 488 (Life Technologies, #A-11029)) diluted 1:500 in blocking buffer for one hour at room temperature. Samples were washed in TBST, counterstained with DAPI, mounted in Prolong Gold and imaged on a Zeiss LSM880 confocal microscope.

**Table 2:** Efficacy of immunostaining protocols with different His and Myc tag antibodies in formalin-fixed paraffin-embedded sections.

Antibody	Dilution	Antigen retrieval condition			
		Citrate	EDTA	Tris-EDTA	Proteinase K
6× His Tag; Mouse (Invitrogen #MA1-21315)	1:200	–	+	–	++
6× His Tag; Rabbit (CST #12698)	1:100	–	–	–	–
Myc Tag; Rabbit (CST #2278)	1:100	+	++	+	–
Myc Tag #2; Mouse (CST #2276)	1:500	+++	+++	+++	–

**–** no signal detected, **+** weak signal detected, **++** moderate signal detected, **+++** strong signal detected.

For wholemount immunofluorescence, FuChi embryos were dissected and fixed in 10% NBF. Samples were washed 3 × 30–60 min in PBS containing 0.5% Triton X-100 (PBTx) with rocking, before blocking in 10% goat serum/PBTx for 2 hours at room temperature with rocking. Samples were then incubated with anti-DAZL antibody (Rabbit (1:250), Abcam, #AB215718) diluted in blocking buffer for 20 hours at 4 °C with rocking. Following primary antibody incubation, samples were washed 5 × 30–60 min in PBTx, before incubation with secondary antibody (Goat anti-rabbit IgG Alexa Fluor 647; 1:200) for 20 hours at 4 °C with rocking. Samples were then washed 5 × 30–60 min in PBTx, counterstained with DAPI (1:2,000 in PBTx) for 1 hour at room temperature, mounted in Prolong Gold and imaged on a Zeiss LSM880 confocal microscope.

Fluorescent in situ hybridisation was performed by hybridisation chain reaction (HCR; Molecular Instruments) [39]. Briefly, FuChi embryos were dissected, and immediately fixed in 4% PFA at room temperature for 1 hour protected from light. Samples were rapidly dehydrated (5 min per dilution series) into 100% methanol, stored at −20 °C for 1–2 hours, before rehydration. HCR was then performed on samples following the manufacturer’s standard protocol using *PTCH1* and *SHH* probes designed and ordered directly from Molecular Instruments. After HCR, embryos were either imaged as wholemounts or processed for cryosectioning; both sample types were imaged using a Zeiss LSM880 confocal microscope.

### Early embryo culture and imaging

Early embryo cultures were performed as in Chapman and colleagues [40]. Briefly, embryos were collected on filter paper, washed in Ringer’s solution to remove excess yolk, and cultured ventral side up in glass-bottom six-well plates or Petri dishes containing a thin layer of albumin/agar (0.3%) mix. Embryos were incubated in a 37 °C imaging chamber, with wet tissue included in the surrounding space and/or unused wells on the plate to ensure humidity, and imaged on a Zeiss LSM880 confocal or Zeiss Axio Zoom.V16 stereo zoom microscope. Drift correction and bleach correction were performed where necessary using Fiji software.

### Fluorescence quality assessment

E3 (HH19) embryos were dissected and immediately imaged (0 hours), and at 72 hours. All images were taken with the same fluorescence settings on a Zeiss Axio Zoom.V16 stereo zoom microscope. Samples were fixed in either 4% PFA or 10% NBF for 1 hour to overnight; all fixation durations were included in the analyses, as we observed no differences between fixation durations. Samples were then transferred into either PBS or 70% ethanol, or alternatively stored in PBS or Ringer’s solution throughout.

### Image-based determination of cell cycle status in cells and tissues

To parameterise an image-based approach to analyse cell cycle status in cells and tissues, we used data from FuChi-derived PGCs. Briefly, following flow cytometry of FuChi PGC lines under different conditions, a sample of PGCs from each line and treatment group was mounted on slides and imaged using a Zeiss LSM880 confocal microscope. Using the Fucci Tools plugin for ImageJ/Fiji [31], fluorescence intensity values were obtained from imaged cultured PGCs (three independently derived lines; three culture conditions) and benchmarked against flow cytometry results from matched samples. Values for all cells were plotted as scatter plots, using min-max normalised fluorescence values for mCherry and mVenus, calculated for each channel as (𝑥 − 𝑥_min_)/(𝑥_max_ − 𝑥_min_), where 𝑥 is the fluorescence value of an individual cell and 𝑥_min_ and 𝑥_max_ are the minimum and maximum fluorescence values of the population, respectively. Using the percentages of PGCs in each cell cycle phase as indicated by flow cytometry analysis, we determined empirical boundaries for G1 (mCherry >0.1, mVenus <0.1), S (mCherry <0.1, mVenus >0.1), and G2/M (mCherry >0.1, mVenus >0.1). Cells below both thresholds (mCherry <0.1 and mVenus <0.1) were classified as double-negative and excluded from cell cycle quantification. These boundaries were then used in all subsequent analyses of cell cycle phase in blastodermal cells, presomitic mesoderm (PSM), neural tube, intestine, and migrating germinal crescent PGCs and their neighbouring somatic cells. Images were acquired in 16-bit format, except for the migrating PGC analyses, which were performed using 8-bit images. Imaging settings were selected to avoid pixel saturation prior to fluorescence quantification.

### Statistical analysis

All statistical analysis was performed using GraphPad Prism 11. For comparing the cell-cycle populations between apical and basal neuroepithelial cells in the neural tube, multiple paired *t*-tests with Holm–Šídák correction were used. For comparison of cell-cycle populations between duodenum and colon tissues, unpaired two-way ANOVAs were performed separately for epithelial, mesenchymal and muscle datasets, followed by Šídák’s multiple comparisons tests. Unpaired *t*-tests, two-tailed and with Welch’s correction, were used to compare E9 and E18 FuChi and wild-type embryo weights. To compare fluorescence levels between fixation and storage conditions, two-way repeated-measures ANOVAs followed by Tukey’s multiple comparisons tests were performed. Details of the specific statistical tests used are also included in the relevant figure legends, along with the number of replicates analysed.

## Results

### Design of the H1.0-Fucci(CA)2 biosensor

To generate a Fucci-expressing chicken line that overcomes the limitations of previously described Fucci systems, we first aimed to design an optimal Fucci biosensor for chicken. Fucci(SA) sensors (Figs 1B, 1C, 2A, and 2B), the most commonly used Fucci pair, incorporate a truncated form of human CDT1(30–120) that undergoes degradation via the SCF^Skp2^ pathway through its Cy motif. While this system functions in mammalian models [12,13,17,41], the Cy motif is not well conserved in non-mammalian species (S1A Fig) and does not function in zebrafish [18], suggesting that it may not function efficiently in chicken. By contrast, Fucci(CA) systems [11] (Figs 1B, 1D, 2A, and 2B), use hCDT1(1–100) with an alternative N-terminal PIP box, which is targeted for degradation via the CUL4^Ddb1^ pathway and is well conserved across species (S1A Fig). The regulation of GMNN, the counterpart in both Fucci pairs, is also highly conserved [18] (S1A Fig). This conservation, combined with the additional advantage of S-phase discrimination offered by the PIP box–based CDT1 sensor, suggested that a Fucci(CA) pair would be the best choice for avian species.

**Fig 2:**
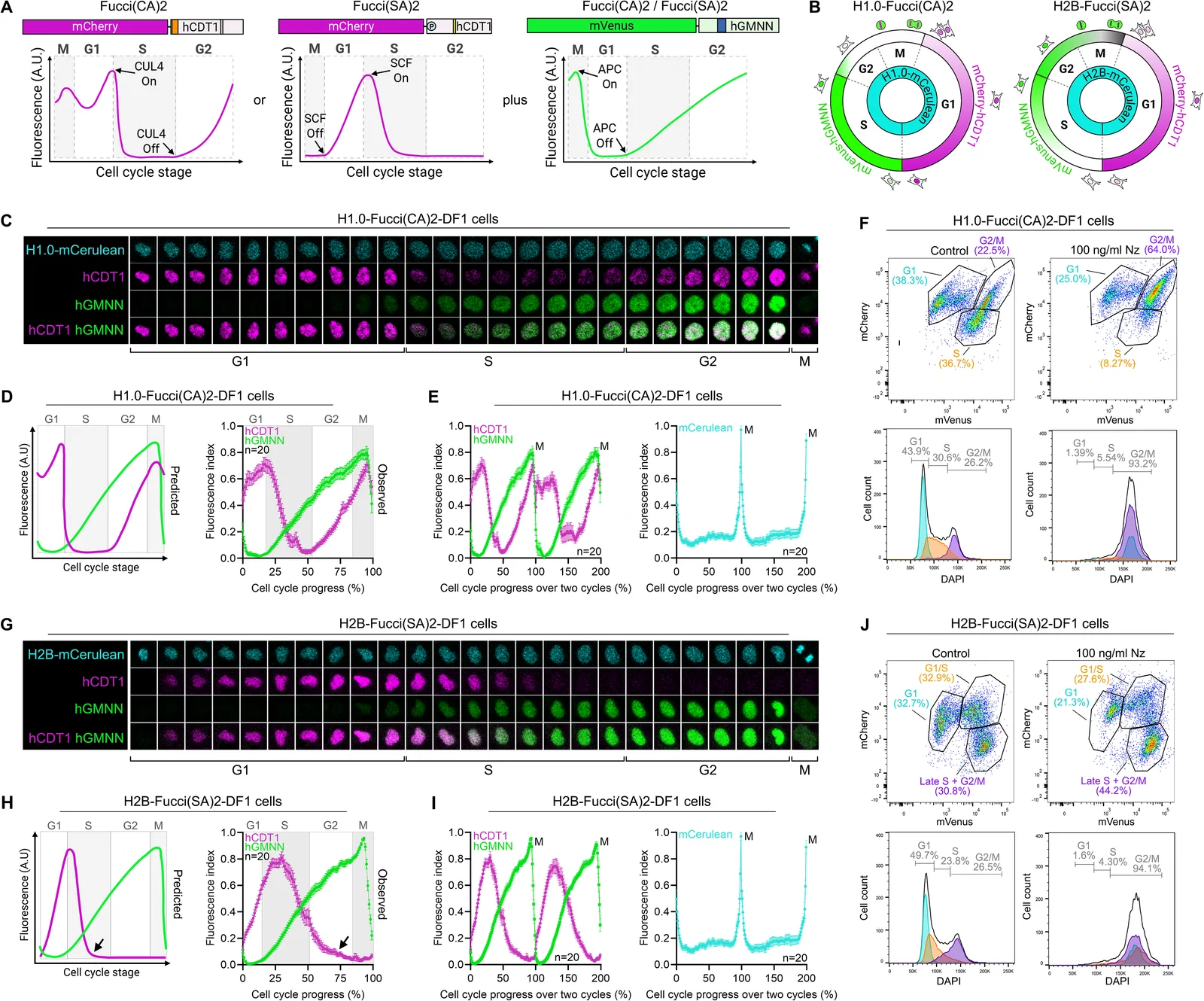
Faithful labelling of G1, S, G2 and M using H1.0-Fucci(CA)2 in chicken cells. **A)** Comparison of the mCherry-hCDT1 fusion proteins used in the Fucci(CA)2 and Fucci(SA)2 systems. The mVenus-hGMNN construct is used in both systems. **B)** Schematic showing the expected fluorescence profiles of the two systems throughout the cell cycle. The Fucci(CA)2 and Fucci(SA)2 constructs also express nuclear H1.0-mCerulean or H2B-mCerulean proteins, respectively, which are present throughout the cell cycle. Note that double-labelled cells are white, negative cells are grey. **C)** Montage following an individual cell and one of its daughters through a complete cell cycle from live cell imaging of stably transduced chicken fibroblasts (DF1) expressing the H1.0-Fucci(CA)2 biosensor. **D)** Mean normalised fluorescence intensity of H1.0-Fucci(CA)2 DF1 cells across the cell cycle. Live cell imaging data from 20 cells, with time normalised such that 0-100% represents one complete cell cycle. **E)** Mean normalised fluorescence intensity of H1.0-Fucci(CA)2 DF1 cells across two consecutive cell cycles. Live cell imaging data from 20 mother-daughter cell pairs, with each cycle normalised to 100% such that 0-200% spans both cycles. **F)** Flow cytometric analysis of H1.0-Fucci(CA)2 DF1 cells in both control conditions and following treatment with nocodazole (Nz), which causes mitotic arrest/G2-M accumulation. Fucci(CA)2 shows faithful correlation between Fucci biosensor fluorescence and DNA content as a proxy for cell cycle stage. **G)** Montage following an individual cell and one of its daughters through a complete cell cycle from live cell imaging of stably transduced chicken fibroblasts (DF1) expressing the H2B-Fucci(SA)2 biosensor. **H)** Mean normalised fluorescence intensity of H2B-Fucci(SA)2 DF1 cells across the cell cycle. Live cell imaging data from 20 cells, with time normalised such that 0-100% represents one complete cell cycle. The observed degradation of hCDT1 is delayed in these cells compared with the expected profile (arrows). **I)** Mean normalised fluorescence intensity of H2B-Fucci(SA)2 DF1 cells across two consecutive cell cycles. Live cell imaging data from 20 mother-daughter cell pairs, with each cycle normalised to 100% such that 0-200% spans both cycles. **J)** Flow cytometric analysis of H2B-Fucci(SA)2 DF1 cells in both control conditions and following nocodazole treatment. Fucci(SA)2 shows less faithful correlation between Fucci biosensor fluorescence and DNA content as a proxy for cell cycle stage in comparison to Fucci(CA)2. Error bars represent standard error of the mean (s.e.m.). Schematics created in BioRender. Glover, J. (2026) https://BioRender.com/37cu02a. The underlying numerical data for this figure is detailed in S1 Data.

Neither Fucci(CA) nor Fucci(SA) discriminate between G2 and M phases and the existing animal models (which are based on Fucci(SA)2) are unable to track cells through early G1 [11]. A tricistronic construct, H2B-Fucci(SA)2, has been described which enabled M-phase discrimination and tracking through early G1 by labelling DNA with an H2B-Cerulean fusion protein [25]. Similarly, the Fucci4 system, although composed of individual constructs and so not useful here, used a fusion with the H1.0 linker histone to achieve the same effect [20]. However, toxicity associated with H2B fusions has also been reported in vivo [42]. Therefore, we selected the H1.0 linker histone fused to mCerulean (optimally spectrally separated from mVenus and mCherry) for use in our chick model. As a final optimisation step, we added HA, 6×His and Myc tags to the H1.0-Cerulean, mCherry-hCDT1(1/100)PIP(+)Cy(−) and mVenus-hGMNN(1/110) components, respectively, to aid in antibody detection in fixed tissues. S1B Fig shows our optimised tricistronic H1.0-Fucci(CA)2 biosensor.

### Faithful discrimination of G1, S, G2 and M phases in chicken cells

We compared the utility of our new H1.0-Fucci(CA)2 biosensor against the previously described H2B-Fucci(SA)2 biosensor [25] by using the piggyBac system [43] to stably integrate each of the biosensors (S1B and S1C Fig) into immortalised chicken DF1 fibroblasts. We found that H1.0-Fucci(CA)2 accurately discriminated cell cycle stage, with similar degradation profiles to Fucci(CA)2 mammalian models [11]. This achieved a clear discrimination between G1, S and G2/M populations and a faithful response to cell cycle perturbations (Figs 2C–2F, S1D, and S1F; S1 Video). However, with H2B-Fucci(SA)2 we identified an extended hCDT1 degradation curve, resulting in persistence of mCherry beyond the G1/S transition (Figs 2G–2J, S1E, and S1F; S2 Video), making cell cycle phase difficult to determine accurately. Both the H2B-Cerulean and H1.0-Cerulean fusions resulted in bright nuclear labelling throughout the cell cycle, including early G1, with sharp peaks in fluorescence intensity as the chromosomes condense in M-phase (Figs 2C, 2E, 2G, and 2I). These results establish H1.0-Fucci(CA)2 as a superior cell cycle probe capable of functionally discriminating all cell cycle phases in chicken cells.

### H1.0-Fucci(CA)2 expressing “FuChi” chickens

We used the piggyBac system to generate stably integrated transgenic PGCs expressing the H1.0-Fucci(CA)2 cell-cycle biosensor (Fig 3A). After transplantation of these PGCs into sterile recipient embryos [29], founder F_0_ males were bred with wild-type Hy-Line hens to generate heterozygous F_1_ (FuChi – “**Fu**cci **Chi**cken”) embryos for analysis (Figs 3A and 3B). FuChi chickens demonstrated fertility, viability and transgene transmission rates of 88%, 92% and 47%, respectively (Fig 3C). FuChi embryos demonstrated strong H1.0-Fucci(CA)2 transgene expression with no observed lethality across all developmental stages (Figs 3B and S2A), were comparable to non-transgenic embryos by visual assessment and by weight as an overall estimate of size (Fig 3D), could be successfully hatched (S2B Fig), and were fertile. Of note, hatched FuChi chicks are initially smaller than wild-type counterparts but do grow at comparable rates (S2C Fig). However, some adult FuChi birds showed poor body condition and required euthanasia, suggesting adverse effects of the H1.0-Fucci(CA)2 transgene and/or consequences of random reporter integration via piggyBac transposition. Mapping H1.0-Fucci(CA)2 integration sites in a pool of FuChi embryos identified insertions on chromosome 1 (NC_052573.1:139,895,885–139,918,586) and chromosome 2 (NC_052574.1:57,700,736–57,701,209). Most embryos analysed carried one of these insertions; however, genotyping of additional embryos indicated that at least one further insertion event was present but was not represented in the sequenced embryo pool (S2D and S2E Fig). We did not observe differences in FuChi reporter expression levels between embryos, regardless of insertion site.

**Fig 3:**
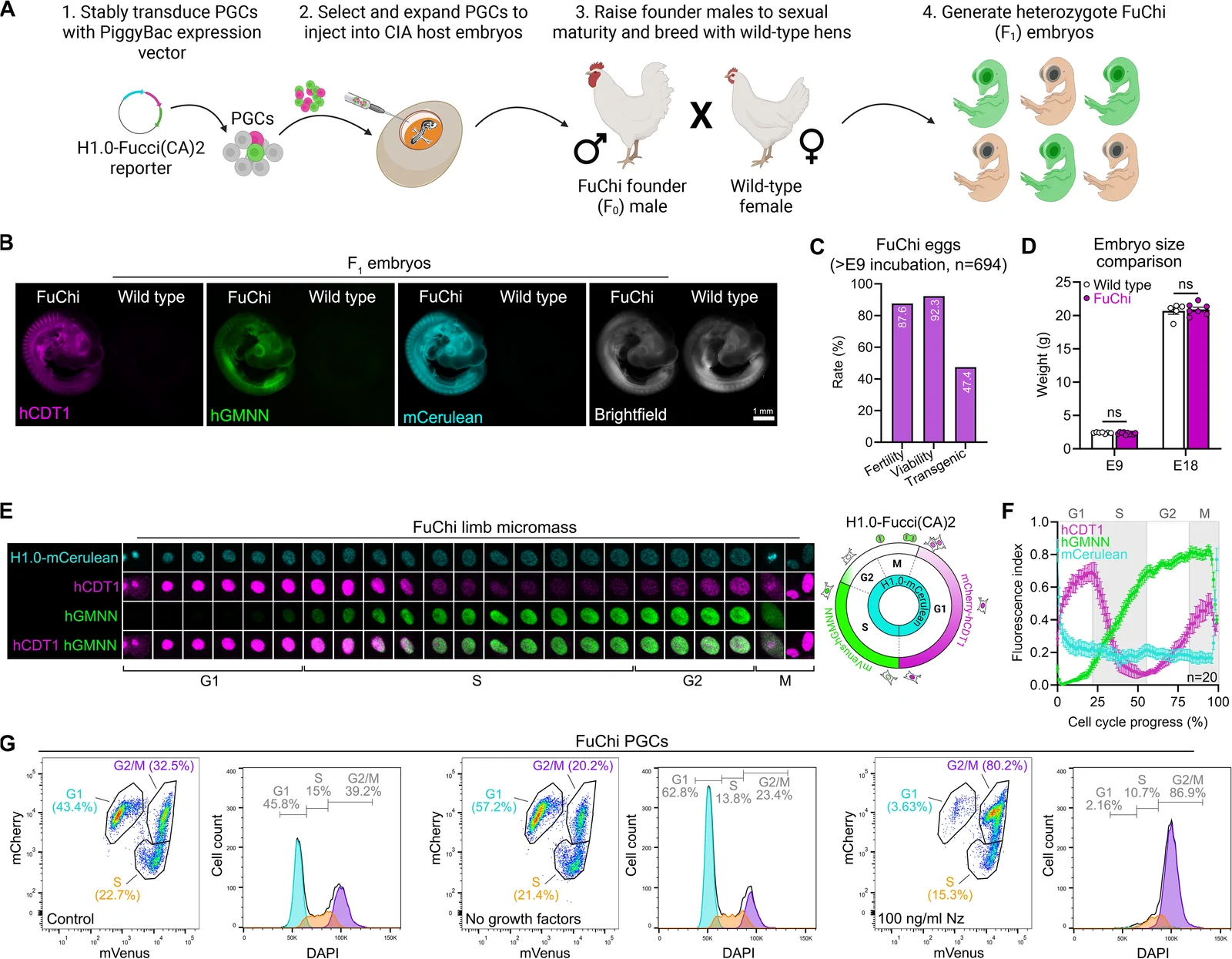
Generation and validation of FuChi reporter chicken. **A)** Schematic of FuChi generation strategy through primordial germ cell (PGC) injection into CIA (chemically induced ablation) sterile surrogate host embryos. **B)** H1.0-Fucci(CA)2 expression in a FuChi F_1_ embryo compared with a wild-type embryo. **C)** Fertility, viability and transgene transmission rates of FuChi surrogate hosts, based on analyses of embryos from embryonic day 9 (E9) onwards. **D)** FuChi embryos develop normally and are comparable in size to wild-type embryos; ns = not significant. **E)** Individual cell montages from limb micromass cultures derived from FuChi embryos with accompanying schematic of H1.0-Fucci(CA)2 reporter profiles. **F)** Mean normalised fluorescence intensity of FuChi limb micromass cells across the cell cycle. Live cell imaging data from 20 cells, with time normalised such that 0-100% represents one complete cell cycle.**G)** Validation of H1.0-Fucci(CA)2 reporter system by flow analysis in PGCs derived from FuChi embryos in both control, growth factor-removed, and nocodazole (Nz)-treated conditions. The reporter profiles accurately reflect DNA content as determined by DAPI staining in both control and experimental conditions. Scale bar = 1 mm. Error bars represent s.e.m. Schematics created in BioRender. Glover, J. (2026) https://BioRender.com/37cu02a. The underlying numerical data for this figure is detailed in S1 Data.

To further investigate the operation of the H1.0-Fucci(CA)2 construct in our FuChi chickens, we derived PGCs and limb micromass cultures from FuChi F_1_ embryos for flow cytometry analysis and confocal imaging, respectively. The temporal fluorescent profiles of the H1.0-Fucci(CA)2 biosensor in micromass cells closely matched those of our H1.0-Fucci(CA)2-DF1 cells (Fig 2D) with clear G1, S, G2/M populations identifiable through Fucci(CA)2 biosensor abundance with labelling of nuclei by H1.0-Cerulean throughout the cell cycle and a strong H1.0-Cerulean peak evident during M-phase (Fig 3E and 3F; S3 Video). Flow cytometry analysis of three independent PGC lines (two male, one female) confirmed the fidelity of the reporter in both control and experimental culture conditions in which cell cycle progression was perturbed (Figs 3G and S2F). Taken together, these results establish FuChi chickens as the first viable stably expressing avian cell cycle biosensor model for in vivo studies.

### Monitoring cell cycle progression throughout chicken embryonic development

Having validated H1.0-Fucci(CA)2 functionality in cell lines derived from FuChi embryos, we next assessed reporter activity during embryonic development. In HH1 embryos, mVenus-hGMNN-positive proliferating cells were abundant throughout the blastoderm, with many cells co-labelled with mCherry-hCDT1 and mVenus-hGMNN, indicating G2/M phase (Fig 4A and 4B). To quantify these patterns, we applied an image-based cell-cycle classification approach benchmarked against flow cytometry of FuChi-derived PGCs (S3 Fig; see Materials and methods). This analysis showed that the majority of blastodermal cells were in either S phase (29.9%) or G2/M phase (58.5%), consistent with diapause-associated cell cycle synchronisation following lower-temperature storage prior to incubation [44]. During gastrulation, primitive streak cells were strongly enriched for mVenus-hGMNN, indicating high proliferation (Fig 4C and 4D), consistent with previous BrdU labelling studies in similarly staged embryos [45]. At the anterior primitive streak, Hensen’s node showed distinct mCherry-hCDT1/mVenus-hGMNN co-labelling, in line with reports that the node contains G2/M-phase cells (Fig 4C) [46]. In contrast, extraembryonic regions, including the area opaca, contained more G1-positive nuclei (Fig 4C). By HH6, static and live imaging showed that the forming notochord and developing extraembryonic regions were enriched for G1 cells, whereas much of the embryo remained mVenus-hGMNN-positive during axis elongation, with more extensive co-labelling in regions such as the head fold and Hensen’s node (Fig 4E and S4 Video). Live imaging of head fold formation (S5 Video) further showed mCherry-hCDT1- and mVenus-hGMNN-labelled nuclei within the forming head fold, indicating that cell-cycle progression and cell movement occur together during head fold morphogenesis. At later stages, reporter signal was detected across embryonic and extraembryonic tissues, with strong mVenus-hGMNN signal marking highly proliferative regions including the tailbud, neural tube and developing brain (Fig 4F).

**Fig 4:**
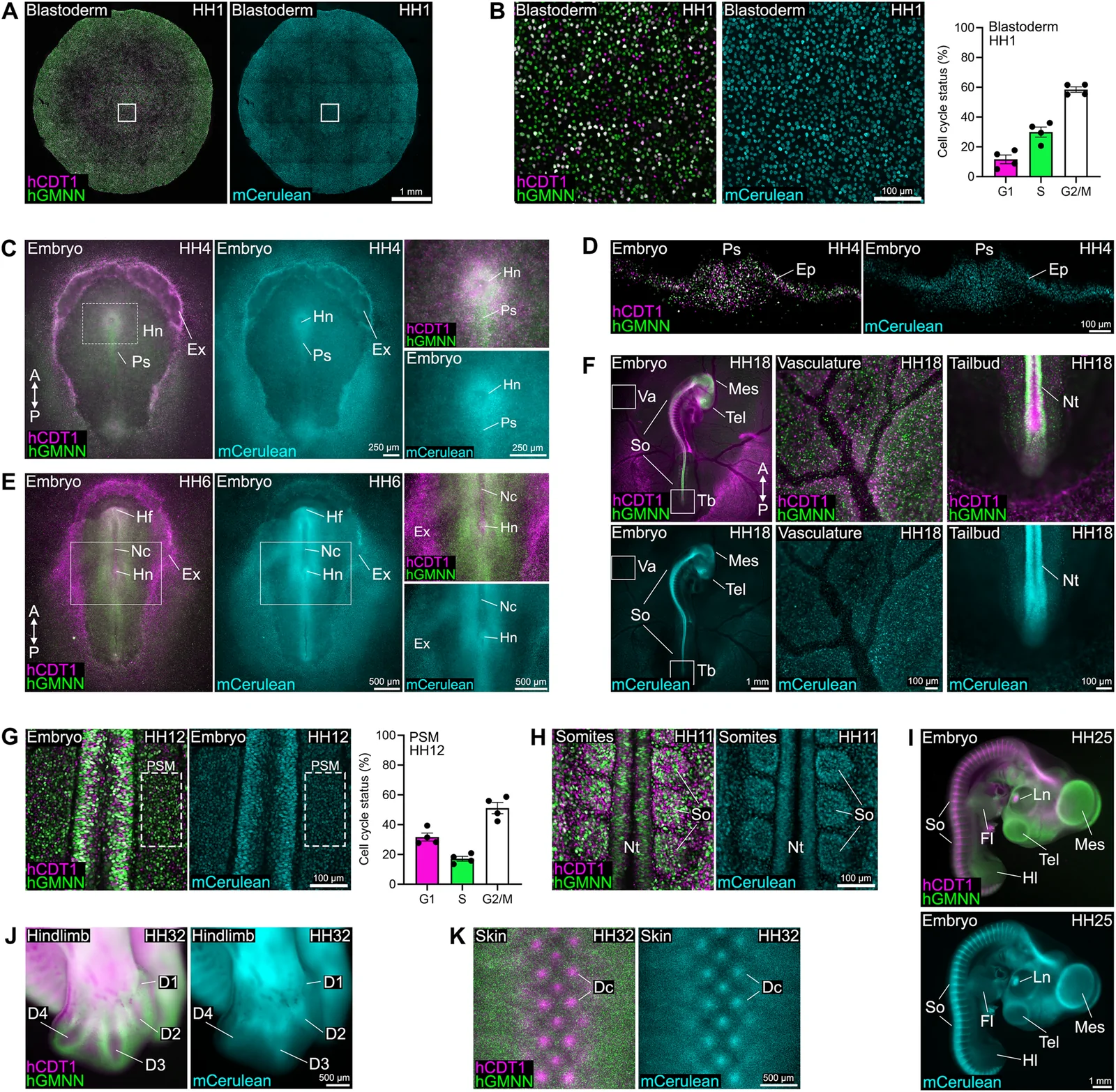
H1.0-Fucci(CA)2 biosensor activity in FuChi embryos during development. **A)** In the blastoderm, mVenus-hGMNN-positive proliferating cells are abundant throughout the embryo. **B)** Higher-magnification imaging of the blastoderm (dashed box in A) reveals that many cells are co-labelled with mVenus-hGMNN and mCherry-hCDT1, indicating G2/M. Image-based quantification of reporter profiles confirms that most blastodermal cells are in G2/M or S phase; *n* = 4 embryos. **C)** In gastrulating HH4 embryos, the primitive streak (Ps) is highly enriched for mVenus-hGMNN compared with the rest of the embryo. Hensen’s node (Hn) contains cells co-labelled with both reporters, indicating G2/M. Extraembryonic (Ex) regions appear less proliferative based on mCherry-hCDT1 signal. **D)** Cryosections show that the primitive streak contains many proliferative cells, based on mVenus-hGMNN and mCherry-hCDT1 reporter profiles, with the epiblast (Ep) also containing many mVenus-hGMNN-labelled nuclei. **E)** In HH6 embryos, the notochord and extraembryonic regions are distinctly G1-enriched/less proliferative, based on mCherry-hCDT1 signal, whereas most other embryonic tissues, including the presomitic mesoderm (PSM), remain largely mVenus-hGMNN-positive. Co-labelled mCherry-hCDT1/mVenus-hGMNN nuclei are evident in Hensen’s node and the developing head fold (Hf). **F)** At HH18, FuChi embryos enable visualisation of circulating blood cells in the developing vasculature (Va) and highlight proliferative regions, including the tailbud (Tb) and developing central nervous system regions, such as the mesencephalon (Mes) and telencephalon (Tel). Somite (So) maturation along the anterior–posterior axis is also apparent. **G)** In the PSM, most cells are in S or G2/M phase, based on visualisation and quantification of reporter profiles in this region (*n* = 4 embryos). **H)** Developing somites, which flank the proliferative neural tube (Nt), contain cells across all cell-cycle phases in both epithelial and mesenchymal compartments, likely reflecting dynamic cellular rearrangements during the mesenchymal–epithelial transition. **I)** By HH25, distinct proliferative domains are apparent. Differentiated somites contain a band of G1 cells, and the lens (Ln) shows a similar abundance of mCherry-hCDT1-positive cells. Elsewhere, the developing forelimb (Fl), hindlimb (Hl), and brain regions appear proliferative/mVenus-hGMNN-positive. **J)** The condensing cartilage of the forming hindlimb digits (D1–4) consists of G1-labelled cells surrounded by a band of mVenus-hGMNN-positive cells. **K)** During feather formation, dermal condensates (Dc) become clearly demarcated from the rest of the skin due to enrichment of cells in G1. The anterior (A) – posterior (P) axis is included for orientation. Scale bars as annotated. Error bars represent s.e.m. The underlying numerical data for this figure is detailed in S1 Data.

Static and live imaging of somitogenesis showed strong mVenus-hGMNN signal in the PSM before somite formation (Fig 4G and S6 Video). Quantification confirmed that most presomitic mesodermal cells were in G2/M (51.2%) or S (17.1%) phase (Fig 4G). As somites formed, proliferation was evident in both the surrounding epithelial cells and the mesenchymal cells undergoing mesenchymal-to-epithelial transition (Fig 4H and S6 Video). During subsequent somite differentiation, mCherry-hCDT1 signal emerged as a central band within each somite. At HH18, this pattern was restricted to more mature anterior somites (Fig 4F), but was present in all somites at later stages (Fig 4I). A similar switch in Fucci reporter profile has been reported in somites of Fucci2 mouse embryos [47]. At mid-developmental stages, limb buds and regions of the developing brain are proliferative, as indicated by mVenus-hGMNN signal, whereas the lens, condensing cartilage of the limb digits, and feather condensates of the skin were enriched for G1 cells (Fig 4I–4K).

We next examined reporter activity across tissue layers and organs, including at later developmental stages. In the neural tube, where cell-cycle-dependent nuclear migration and mitoses at the ventricular surface of the neuroepithelium are well characterised [48], G1 was the largest cell-cycle fraction in both apical and basal neuroepithelial cells, with a higher proportion in basal cells (73.2%). Conversely, apical cells showed relative enrichment for S phase (27.9%) and G2/M (24.9%) compared with basal cells (17.2% S phase; 9.6% G2/M) (Fig 5A). Using H1.0-mCerulean to identify mitotic cells, we observed divisions oriented perpendicular to the dorso-ventral neural tube axis (Fig 5B), as previously described [49,50]. These mitotic cells also showed the expected cytoplasmic mVenus-hGMNN signal during M phase (Figs 1D, 3E and 5B).

**Fig 5:**
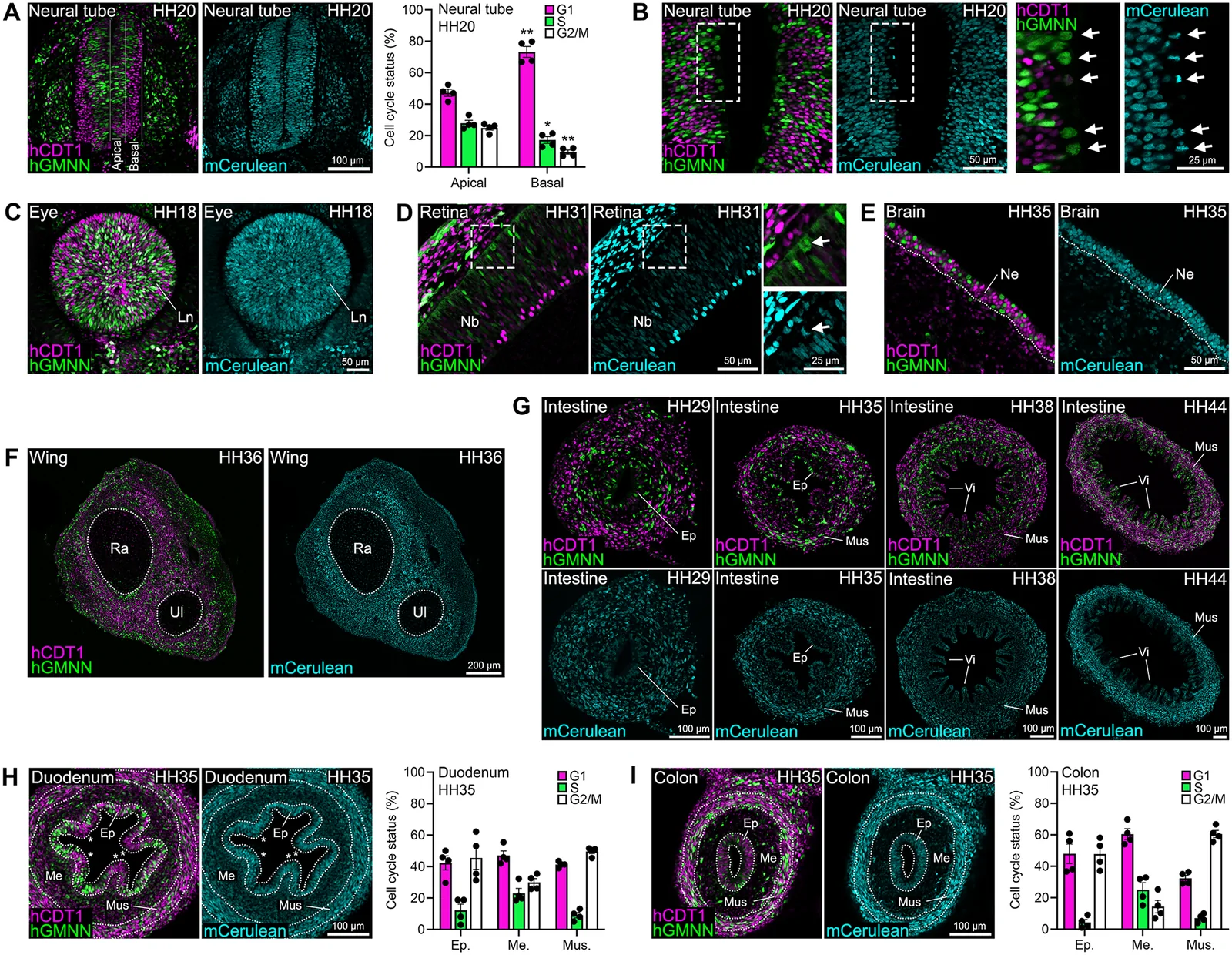
Analysis of cell-cycle profiles in developing tissues of FuChi embryos. **A)** Cryosections of the neural tube show clear demarcation of cell-cycle status. Image-based analysis revealed apical cells of the neuroepithelium showed a higher proportion of S and G2/M than basal cells, whereas basal cells were predominantly mCherry-hCDT1-positive, indicating G1. *p < 0.05, **p < 0.01; paired *t* tes*t*, *n* = 4 embryos. **B)** Dividing mVenus-hGMNN-positive cells at the apical surface of the neuroepithelium, indicated by arrows, predominantly divide perpendicular to the dorso-ventral axis of the neural tube. **C)** At HH18, cells at the centre of the lens (Ln) show a more proliferative profile than those at the periphery. **D)** In the retina, cells often divide perpendicular to the local retinal surface, as illustrated by the mVenus-hGMNN-positive mitotic neural retinal cell of the neuroblastic layer (Nb), adjacent to the retinal pigment epithelium, indicated by the arrow in the magnified dashed-box region. **E)** Cryosection of a FuChi embryonic brain showing proliferation restricted to the neuroepithelium (Ne). **F)** Cryosection of an HH36 wing showing the radius (Ra) and ulna (Ul) surrounded by mCherry-hCDT1-positive developing musculature. **G)** Cryosections of FuChi intestine across developmental stages. During villus (Vi) formation, proliferating mVenus-hGMNN-labelled epithelial cells become localised to basal regions of the luminal epithelium (Ep) corresponding to the future intervillous/villus base region. The muscle (Mus.) layers initially contain both mVenus-hGMNN- and mCherry-hCDT1-labelled nuclei, before becoming predominantly mCherry-hCDT1-positive/G1-labelled at later stages. **H)** Analysis of the cell-cycle profile of the duodenum at HH35 reveals local restriction of mCherry-hCDT1/G1 labelling to the tips of the luminal ridges in the epithelium (Ep), indicated by asterisks, with mVenus-hGMNN-positive cells present only at the interridge base. At this stage, the intestinal mesenchyme (Me) and muscle layers (Mus) are also actively proliferating, as determined by image-based quantification of cell-cycle status (n = 4 embryos). **I)** Analysis of FuChi embryonic colon shows an overall reduction in proliferation among mesenchymal cells compared with the duodenum, but an increase in co-labelled G2/M nuclei in the muscle layers at this stage as determined by image-based quantification (*n* = 4 embryos). Scale bars as annotated. Error bars represent s.e.m. The underlying numerical data for this figure is detailed in S1 Data.

In the HH18 eye, the developing lens showed a mixed reporter profile, with mVenus-hGMNN-positive cells concentrated centrally and mCherry-hCDT1-positive cells enriched at the periphery (Fig 5C), similar to previous analyses at comparable stages [51]. Cryosections of FuChi retina showed a high proportion of cells in S and G2/M, consistent with the retina being a highly proliferative tissue during vertebrate development [52]. Mitotic figures were often oriented perpendicular to the retinal surface (Fig 5D), consistent with previous data [53]. In other tissues examined at mid-developmental stages, we found the neuroepithelium of developing brain regions displayed high levels of proliferation as previously described (Fig 5E) [54], while the bones of the wing and surrounding muscle contained mostly cells in G1 (Fig 5F).

Finally, we analysed cell cycle profiles in the developing intestine of FuChi embryos across multiple stages (Fig 5G). Before villus formation begins, mVenus-hGMNN-positive proliferating cells are distributed throughout the epithelium and surrounding mesenchyme of the HH29 intestine. As development progresses, luminal ridges transition through a zig-zag phase to form villi, shaped by the surrounding muscle layers [55]. While mVenus-hGMNN-positive cells are present at HH35, by HH38 the muscle layers are predominantly mCherry-hCDT1-positive, indicating enrichment for cells in G1. In the epithelium, proliferative cells become restricted to the villus bases/intervillous regions at later stages, a consistent occurrence across species thought to confine the presumptive stem cell populations to this region [55–57] (Fig 5G). The mesenchyme contains a mixture of mCherry-hCDT1- and mVenus-hGMNN-labelled nuclei across stages (Fig 5G).

We then compared the duodenum and colon in HH35 FuChi embryos. In chicken, the small intestine will eventually form villi (Fig 5G and 5H), whereas the large intestine forms smaller-scale structures known as cuffs (Fig 5I) [58]. Although the overall reporter profiles were similar, cell-cycle distribution differed significantly between duodenum and colon in the mesenchyme (*p* = 0.0019) and muscle layers (*p* < 0.0001), but not in the epithelium. Specifically, the mesenchyme of the duodenum contained fewer cells in G1 than the colonic mesenchyme (47.0% versus 60.5%; *p* = 0.038) and more cells in G2/M (30.0% versus 14.4%; *p* = 0.014). In contrast, the muscle layers of the duodenum contained more cells in G1 than the corresponding layers of the colon (41.3% versus 32.4%; *p* = 0.0038) and fewer cells in G2/M (49.6% versus 60.6%; *p* = 0.0006). Most notably, epithelial cells at the tips of the forming luminal ridges in the duodenum showed spatially restricted mCherry-hCDT1 signal (Fig 5H; observed in 4/4 intestines analysed), whereas cells at the interridge base were often mVenus-hGMNN-positive. Although similar to the later stage profile (Fig 5G), this pattern suggests that the spatial restriction of proliferation begins several days earlier than previously reported [55].

Together, these results show that the H1.0-Fucci(CA)2 biosensor intensity profiles in FuChi embryos recapitulate known tissue-specific cell cycle patterns and can reveal unrecognised proliferative organisation during chicken embryogenesis.

### Robust H1.0-Fucci(CA)2 stability in fixed tissues and immunodetection after paraffin embedding

Next, to assess the stability and usability of H1.0-Fucci(CA)2 in FuChi tissues, we analysed persistence of the fluorescence signal following common methods of fixation and tissue preparation (S4 Fig). H1.0-Fucci(CA)2 signal was stable following a range of fixation methods compared to unfixed specimens (S4A and S4B Fig), with only a slight increase in autofluorescence observed in the fixed samples (S4B Fig).

While H1.0-Fucci(CA)2 signal can be directly observed in cryosections and fixed wholemount tissue (Figs 4 and 5), we wanted to test whether our reporter embryos could be combined with gene and protein expression analysis. We performed fluorescent in situ hybridisation to detect expression of the hedgehog target gene *PTCH1* in the developing embryo (Fig 6A). *PTCH1* is expressed in the neural tube, limb bud, and somites, with expression closely correlating with proliferating cells in the neural tube (Fig 6A) [59]. Conversely, *SHH* expression, which induces *PTCH1* expression, is restricted to the notochord and floor plate of the neural tube (Fig 6B) [60]. Immunostaining of FuChi embryonic spinal cord cryosections for YAP1 (Fig 6C) revealed YAP1 protein expression and localisation in the progenitor zone, a region associated with high proliferation and known to be regulated by YAP [61]. Additionally, the inclusion of epitope tags in H1.0-Fucci(CA)2 enabled detection of the reporters by immunofluorescence in formalin-fixed paraffin-embedded (FFPE) sections (Figs 6D and S5). The reporter profiles of immunostained FFPE sections were comparable to those seen in cryosections across several tissues, including the brain (Figs 5E, 6D, and S5A), intestine (Figs 5G–5I, S5B and S5D) and neural tube (Figs 5A, 6A–6C, S5C, and S5E).

**Fig 6:**
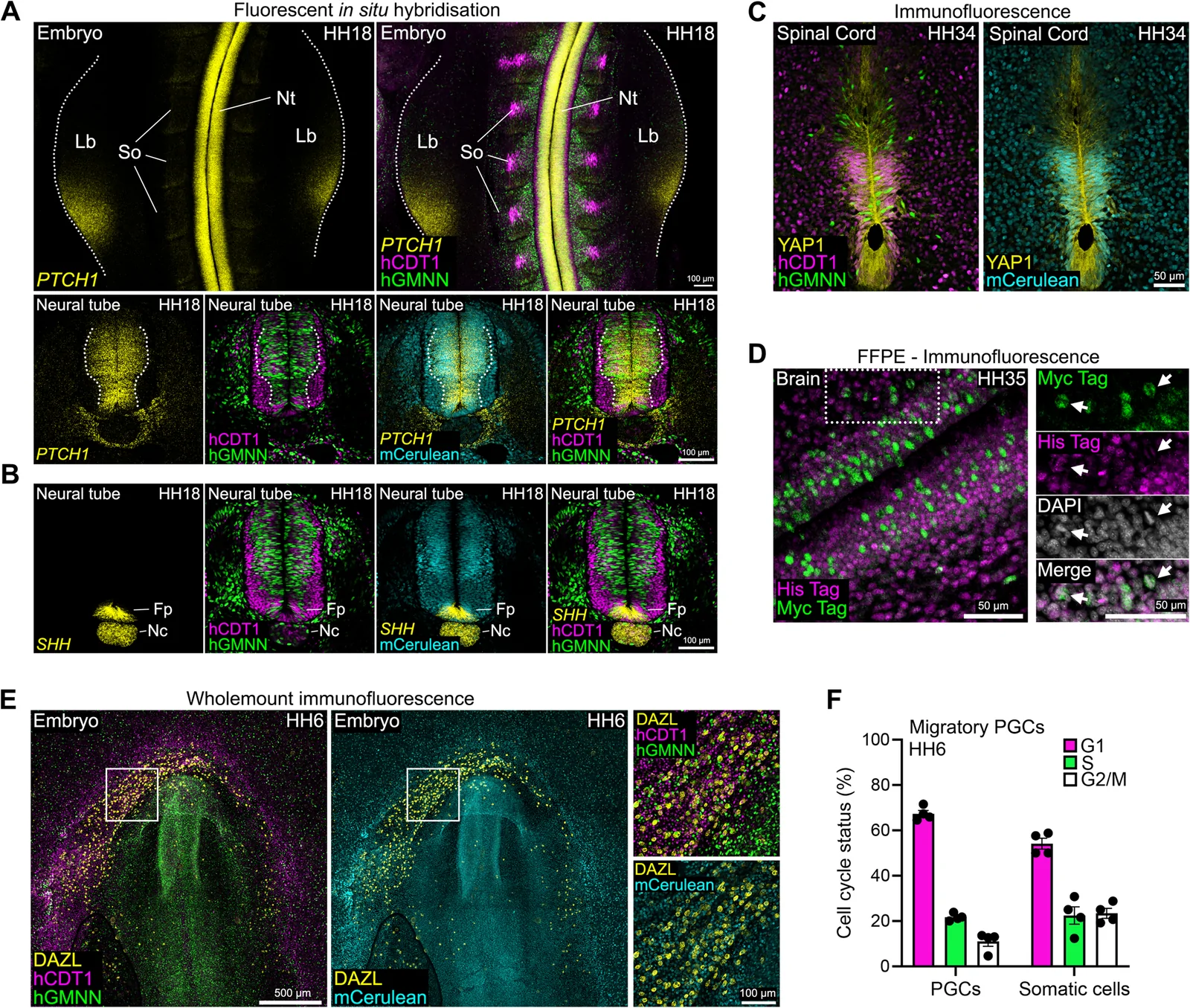
Combining cell cycle status with detection of endogenous protein and mRNA expression. **A)** Hybridisation Chain Reaction (HCR) to detect *PTCH1* transcript expression in HH18 FuChi embryos. Top panels show wholemount staining and bottom panels are cryosections of these embryos. Strong *PTCH1* mRNA expression can be seen in the neural tube (Nt; expression domain demarcated by dashed lines), corresponding to a region of high proliferation, in the limb bud (Lb), and with weak expression in the somites (So). **B)** Expression of *SHH*, which induces *PTCH1* expression, is restricted to the floor plate (Fp) and the notochord (Nc). Fluorescent reporter signal remains robust following HCR. **C)** Immunofluorescent detection of total YAP1 protein in a cryosection of the developing spinal cord of a HH34 FuChi embryo. **D)** Detection of the FuChi epitope-tagged reporters, 6×His-mCherry-hCDT1 (His Tag) and Myc-mVenus-hGMNN (Myc Tag), in formalin-fixed paraffin-embedded (FFPE) brain tissue. Myc tag-positive/mVenus-hGMNN-positive proliferative cells are abundant in the neuroepithelium, whereas the underlying non-neuroepithelial tissue is predominantly His tag-positive/mCherry-hCDT1-positive, indicating G1. These patterns are comparable to cryosections (Fig 5E), with staining further validated by His tag-labelled mitotic cells shown in magnified images of the region within the dashed box. **E)** Detection of FuChi reporter signal in migrating PGCs, identified by wholemount immunofluorescence for the germ cell marker DAZL, as they move anteriorly towards the germinal crescent. **F)** Percentage of PGCs and neighbouring somatic cells in each cell cycle phase determined from confocal images. Between 55 and 172 cells of each type were analysed per embryo (*n* = 4 embryos). Scale bars as annotated. Error bars represent s.e.m. The underlying numerical data for this figure is detailed in S1 Data.

To further explore the utility of FuChi embryos, we tested whether it was possible to assess the proliferation profile of native migrating cells using immunofluorescence on wholemount embryos (Fig 6E). In chickens, PGCs migrate anteriorly from the centre of the blastoderm, ahead of the forming embryo to populate the germinal crescent during early development. Here, they enter the bloodstream and are translocated to the anterior vitelline veins where they exit the bloodstream and migrate into the genital ridges, before finally populating the gonads [62,63]. However, the proliferative status of PGCs during their initial migratory steps, prior to entering the bloodstream, is unknown. Using our imaging-based cell cycle quantification approach, we found that as PGCs migrate towards the germinal crescent the majority of cells reside in G1 (67.3%), compared to those in S (21.7%) and G2/M (11.0%) (Fig 6F). This differs from both neighbouring somatic cells (Fig 6F) and also from PGCs in culture (Figs 3G, S2F, and S3) indicating that, unlike the rapidly proliferating germ cells in the gonads [64], PGCs at these initial migratory stages are not undergoing frequent cell division.

### Real-time visualisation of cell cycle dynamics during gastrulation

A major attraction of Fucci systems is that they can be used to image cell cycle progression, not only in isolated live cells, but also in complex tissues and even whole embryos. Control of the cell cycle plays an important role in the embryonic development of all organisms, including chick. However, so far there is only limited information available on cell cycle changes during avian development. To assess the potential of the H1.0-Fucci(CA)2 system for live imaging we used light sheet microscopy to monitor cell cycle changes from the onset of development after egg laying (Eyal-Giladi and Kochav (EG and K) [65] stage XII) to the primitive streak stage (HH4) (Fig 7A and S7 Video). Embryos were imaged at 10x magnification, allowing visualisation and detection of mVenus (hGMNN) and mCherry (hCDT1) in all nuclei of the surface layers of the embryo at 8–10 min time intervals. To quantitatively assess changes in the distribution of cells in different cell cycle phases during the early stages of development, we measured the total number of nuclei detected in G1, S, and G2/M based on mVenus-hGMNN and mCherry-hCDT1 fluorescence as a function of time in both the embryonic and extraembryonic territories (Fig 7B). This revealed that at the onset of development most cells are in S phase and progress into G2 followed by mitosis and G1 in a partially synchronised manner which persists in a further subset of cells during primitive streak formation (Fig 7B). Next, to characterise the temporal cell cycle dynamics in vivo we measured the kinetics of fluorescence changes in individual cells in the embryonic and extraembryonic regions at the onset of development (Fig 7C, S8–S10 Videos). This analysis revealed that the cell cycle is around 10 hours in both territories (Fig 7D; representative of four independent repeats). The synchronised manner of cells progressing from S through G2 followed by mitosis to G1 from the onset of development was also apparent in the global analysis of cells in the specific regions analysed (S6A Fig).

**Fig 7:**
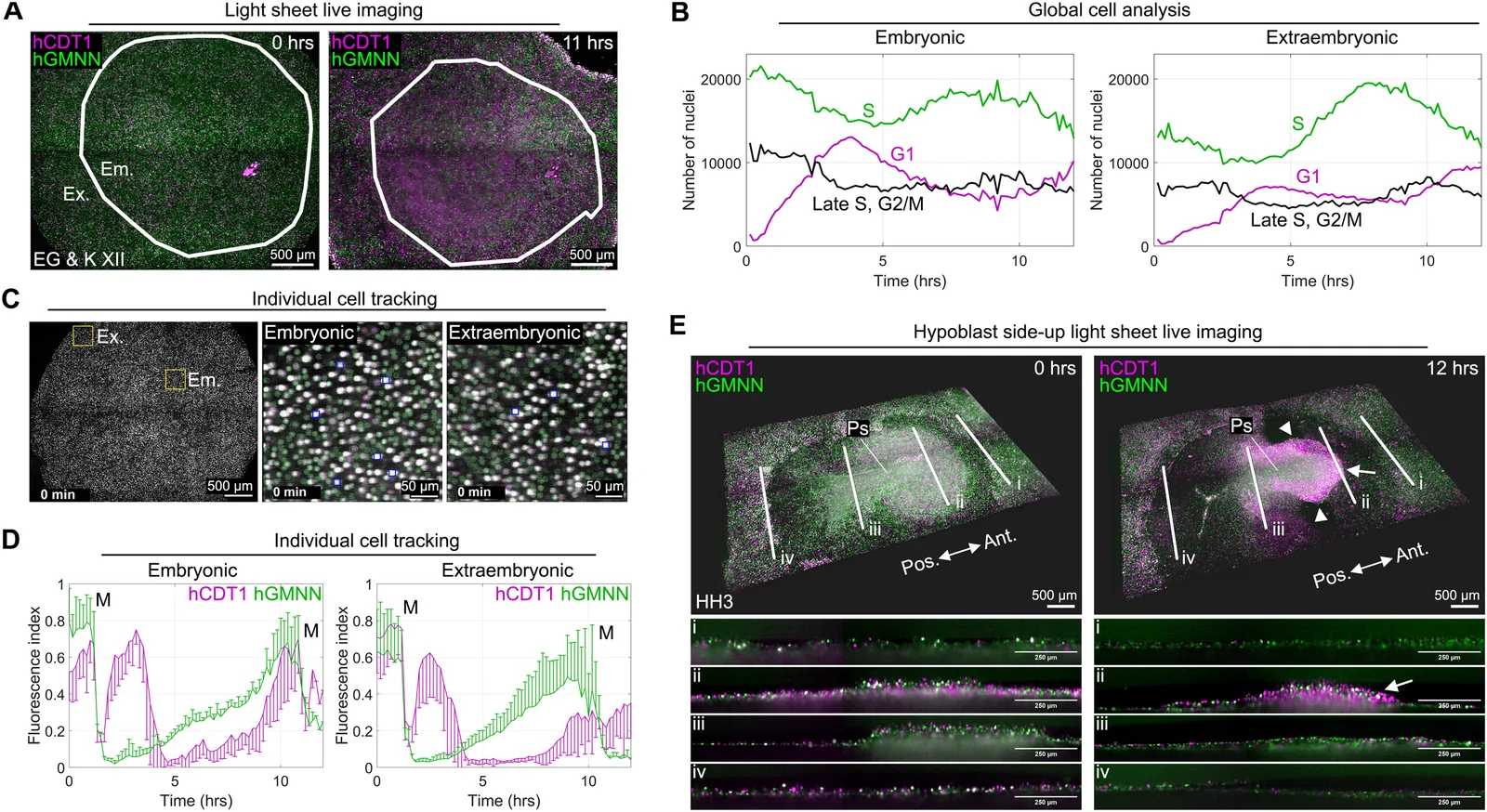
Visualisation and assessment of cell cycle dynamics during early embryonic development. **A)** Epiblast surface views from a representative light sheet experiment performed on a FuChi embryo at the start and after 11 hours of development. The boundary separating the embryonic (Em.) and extraembryonic regions (Ex.) is indicated by the white line, calculated as described in Materials and methods. **B)** Global cell counts of embryonic and extraembryonic regions of the FuChi embryo visualised by light sheet microscopy across time in Fig 7A. All cells detected in the analysed regions were categorised based on their fluorescence profile at the time interval; mCherry-hCDT1-positive (G1; magenta), mVenus-hGMNN-positive (S; green), or positive in both channels (Late S, G2/M; black). The same observations were made across four independent light sheet experiments. **C)** Left panel: Representative image of epiblast surface view from light sheet time-lapse imaging of a FuChi embryo with regions of interest in the embryonic and extraembryonic regions highlighted (yellow boxes). Right panels: Individual cells, indicated by blue squares, were tracked in the embryonic and extraembryonic regions (n = 4 and 6 cell tracks, respectively). Images represent a single time point from a light sheet time-lapse video. **D)** Mean normalised fluorescence profiles of individual cells tracked in the embryonic and extraembryonic cut-out regions highlighted in (C). Error bars represent standard deviation. **E)** 3D hypoblast view of a developing FuChi embryo at two successive time points 12 hours apart. Four optical sections (i-iv, panels below), taken along the anterior (Ant.) to posterior (Pos.) plane and including extraembryonic (i) and embryonic regions (ii, iii), are also shown at the positions indicated by white lines. Note that cells egressing from the primitive streak (Ps; arrowheads), and cells of the prechordal plate and anterior mesoderm that have already migrated away (arrow), are predominantly in G1. Scale bars as annotated. The underlying numerical data for this figure is detailed in S1 Data.

Finally, the epiblast view showed that during primitive streak formation many cells in the deeper layer of the embryo resided in G1 while migrating away from the streak (S6B Fig). To investigate this in more detail, we performed hypoblast side-up experiments (Fig 7E and S11 Video), which allow better viewing of hypoblast cells and cells emerging from the streak after their ingression. Strikingly, these experiments (three independent repeats) revealed that mesendoderm cells emerging from the primitive streak are in S phase, transition through G2/M, and enter G1 during their migration away; this is particularly evident in cells forming the prechordal plate and anterior mesoderm (Fig 7E and S11 Video). These whole embryo live imaging results demonstrate that the H1.0-Fucci(CA)2 system allows tracking of cell cycle progression in vivo for prolonged periods of time. Importantly, it highlights the dynamic cell cycle changes that cells undergo during key morphological events such as gastrulation. The significance of these findings, including the extent to which cell cycle progression influences cell fate, will be elaborated in more detail in future experiments on FuChi embryos.

## Discussion

Here, we describe H1.0-Fucci(CA)2, a novel tricistronic cell cycle biosensor capable of resolving G1, S, G2 and M phases from a single construct. Furthermore, we describe FuChi, the first avian cell cycle reporter model, and demonstrate the functionality of the H1.0-Fucci(CA)2 biosensor in chickens in vivo. Fucci(CA)2 is functional in chicken, both in vivo and in vitro, and displays a fluorescent reporter profile consistent with that observed in mammalian systems [11]. A major advantage of this reporter is its ability to distinguish between S and G2/M phases—a key improvement over the original Fucci(SA)2 system. In addition, the inclusion of H1.0-mCerulean allows for continuous cell tracking throughout the cell cycle and enables specific identification of M-phase via labelling of mitotic chromatin. The incorporation of epitope tags further expands the system’s utility by facilitating analysis in fixed tissues.

Although Fucci(SA)2 systems using human CDT1 fusion proteins have previously been used in chicken cells in vitro and through transplantation in vivo [23,24], our results suggest that in this system human CDT1 is not efficiently degraded during S phase, thereby reducing the overall fidelity of the Fucci(SA)2 reporter in chick. This likely arises from differences between the core residues of the Cy motif in mammalian (Arg, Arg, Leu) and avian (Lys, Arg, Leu) CDT1, which are essential for SCF-mediated CDT1 degradation [5,66,67]. This is consistent with reports that human CDT1-based Fucci reporters requiring SCF-mediated degradation are not functional in zebrafish [18]. The Fucci(CA)2 system overcomes species differences in the Cy motif by utilising the PIP box of CDT1, a feature conserved across metazoans [68]. As hGMNN degradation is well conserved and functional between species [18], our data suggest that the pre-existing human Fucci(CA)2 reporter system will function in other model organisms, without the need for the generation of new species-specific CDT1 variants.

The ability to analyse cell cycle status in vivo in real-time and in fixed sections enables many downstream applications. Here, we investigated the proliferative status of migrating PGCs during early embryonic development in vivo. Previous studies analysed chicken PGC cell cycle status either in culture [24,69] or from stages beyond E2.5 [64,70], at which point the PGCs have entered the bloodstream. Our results reveal that at HH stage 6, as PGCs migrate anteriorly to the germinal crescent and prior to entering the bloodstream, the majority of cells are in G1 (~70%). A potential reduction in cell division before entering the vasculature is consistent with PGC number not rapidly increasing across these stages [63], as well as PGC cultures derived from EG and K stage X embryos showing reduced proliferation compared to those derived from circulating PGCs [69]. This suggests a trade-off between proliferation and active migration in PGCs at this stage, distinct from earlier passive embryo-wide movements or later circulation via the bloodstream [71]. Beyond germ cell development, FuChi embryos could be used to interrogate the proliferative status of other migrating cells such as neural crest cells, leveraging the chick embryo’s well-established advantages for neural crest research [72,73].

The cell cycle duration observed in the embryonic and extraembryonic tissues here is faster than that measured in tissue culture and is consistent with previous reports from tracking experiments in gastrulating embryos [32]. The results show that most cells are in S or G2/M phase during the diapause that follows egg laying. Upon re-entering development, cells progress through S and G2/M, resulting in the observed partial synchrony of later cell divisions. The results also suggest that cells are in S phase while emerging from the primitive streak and transition to G1 as they migrate away from the streak, which is particularly prominent for cells forming the anterior and prechordal plate mesoderm. The significance of this pattern and its link to differentiation could be elucidated by detailed cell tracking at higher magnification in future work.

Our finding that the chick PSM is highly proliferative is consistent with previous studies showing that mitotic activity is greatest in cells shortly before they undergo somitogenesis [74]. Together with heat-shock experiments that produced temporally distinct somite defects, these observations contributed to the proposal of a cell-cycle clock model of somite segmentation [75]. Although we did not quantify cell-cycle phase along the anterior-posterior axis of the PSM, we observed no obvious regional differences in reporter labelling, suggesting that proliferating cells are distributed throughout the PSM, as has been reported in mouse [76] and zebrafish [77]. Whether elevated proliferation in the chick PSM directly contributes to somitogenesis remains to be determined. One possibility is that morphogen signalling is modulated during specific phases of the cell cycle, thereby influencing signalling oscillations, as recently described in mouse [78]. FuChi embryos offer an ideal system to dissect this in future, combining live-imaging capability with compatibility for downstream applications such as in situ hybridisation to capture gene expression profiles.

Developing Fucci-expressing cell lines requires strategies that achieve stable integration of the construct at moderate expression levels with balanced stoichiometry of the fluorescent reporters. Previously, this was achieved using the Flp-In system [15,79]. In this study, we successfully applied piggyBac transposon technology in the chick, consistent with recent results in human cells [25]. This approach achieves comparable integration without the need for prior genomic insertion of a Flp-In cassette or reliance on viral transduction. As such, the H1.0-Fucci(CA)2 piggyBac construct described here should enable straightforward generation of Fucci reporter lines in diverse systems including primary cells, organoids, and cancer cell models across mammalian and avian species, as well as alternative animal models such as mouse and zebrafish.

The chick embryo is a powerful model system that has been instrumental in advancing our understanding of vertebrate development and disease since the 17th century [80]. Over the past two decades, progress in chicken stem cell and transgenic technologies [27,29,81,82] has enabled the generation of several fluorescent reporter and gene knockout chicken lines, leading to important discoveries in embryonic development, human congenital disorders, and infectious diseases [35,83–87]. The growing repertoire of transgenic tools, now including the FuChi line, is helping to establish the chicken as a highly tractable alternative to more commonly used models such as the mouse and zebrafish. Furthermore, the chorioallantoic membrane (CAM) of FuChi embryos will provide a platform to investigate host tissue proliferation and dedifferentiation in response to human or animal xenografts (tumours or cell lines) or viral infection, and to evaluate potential therapies in a live, vascularised microenvironment [88].

From a 3Rs (Replacement, Reduction and Refinement) perspective, the chick embryo offers clear ethical and logistical advantages. For example, in both the United Kingdom and European Union, embryos up to embryonic day 14 are not classified as protected animals, and their use does not require the sacrifice of a pregnant female. This supports Reduction in the number of protected animals, as a single small transgenic flock can generate ~1,300 embryos annually [89]. The chick also contributes to Replacement of early-stage mammalian models and allows for Refinement through non-invasive *in ovo* imaging and precise manipulation during development.

## Limitations

While the FuChi system offers comprehensive resolution of all major cell cycle phases (G1, S, G2, and M) and enables high-resolution live imaging of proliferation during early avian development, several limitations remain. First, although reporter activity is strong and widespread in early FuChi embryos, its performance in later developmental stages has not yet been systematically evaluated. Differences in chromatin environment, epigenetic silencing, or protein turnover across tissues and timepoints could affect biosensor performance at later stages. Second, although FuChi embryos develop normally, some adults showed deleterious effects, likely due to piggyBac-mediated insertion-site variability, highlighting the need for a chicken safe-harbour locus to enable ubiquitous expression while minimising insertional risk. Third, as previously reported, the Fucci(CA) probes rely on the CUL4^Ddb1^-mediated degradation pathway; therefore, the intensity of the hCDT1(1/100)PIP(+)Cy(−) signal may be reduced in contexts where DNA damage is induced (e.g., by UV irradiation). Fourth, although Fucci(CA)2 distinguishes G1 from S, and S from G2/M, and the mCerulean-tagged histone H1.0 enables visualisation of mitotic chromatin, precise quantification of transitions (e.g., G2 to M) may still require complementary temporal data or additional markers, particularly in highly dynamic contexts. Finally, although the FuChi biosensor enables clear phase discrimination, analysing proliferation in rapidly morphing or migrating tissues (e.g., the primitive streak or neural crest streams) may require high-frame-rate imaging or integration with lineage markers to avoid misinterpreting positional shifts as cell-cycle transitions. This will be enabled by application of the latest AI-driven segmentation and tracking algorithms [90,91].

## Conclusions

In conclusion, the FuChi transgenic line provides a powerful and versatile tool for real-time analysis of the cell cycle in the chicken embryo, both in vivo and in vitro, across multiple developmental stages. We show that the Fucci(CA)2 system functions robustly in the avian context, offering precise discrimination of all major cell cycle phases, including mitosis, while the addition of H1.0-mCerulean and epitope tags enhances both live imaging and fixed-tissue analysis, including in FFPE samples. The stability and accessibility of this reporter line make it a valuable resource for researchers worldwide. Collectively, these advances position FuChi embryos as a premier in vivo system for investigating developmental cell-cycle kinetics, with clear technological advantages over current mouse models. We anticipate that FuChi will advance studies in development, growth, tissue homeostasis, and disease. FuChi eggs will be made available upon request from the National Avian Research Facility at the Roslin Institute, UK.

## Supporting information

S1 Fig Comparison and validation of Fucci reporter systems in chicken cells in vitro. **A)** Comparison of the PIP box (targeted by CUL4^Ddb1^) and Cy motif (targeted by SCF^Skp2^) degradation domains of CDT1 and the destruction box (targeted by APC^Cdh1^) of GMNN across vertebrate species. While the destruction box of GMNN is well conserved in mouse, human and chicken, as are the core residues of the PIP box (Ψ represents any moderately hydrophobic amino acid (L, V, I, or M); Ω is an aromatic residue (Y or F)), the Cy motif of CDT1 is not well conserved across species. B and C) Design of piggyBac Fucci reporter constructs. **B)** The H1.0-Fucci(CA)2 reporter cassette uses a CAG promoter to drive expression of H1.0-mCerulean, mCherry-hCDT1(1/100)PIP(+)Cy(−), and mVenus-hGMNN(1/110) fusion proteins, each containing an epitope tag (HA, 6× His, or Myc), and which are upstream of the puromycin resistance gene. **C)** The H2B-Fucci(SA)2 reporter cassette expresses H2B-mCerulean, mCherry-hCDT1(30/120)PIP(−)Cy(+) and mVenus-hGMNN(1/110) fusion proteins, upstream of the puromycin resistance gene. ITR = Inverted terminal repeats. The presence or absence of the PIP domain and Cy motif of human CDT1 proteins are denoted in parentheses. **D)** Extended time-lapse images showing two full cell cycles of H1.0-Fucci(CA)2 expressing DF1 cells. **E)** Extended time-lapse images showing two full cell cycles of H2B-Fucci(SA)2 expressing DF1 cells. **F)** Comparison of Fucci(CA)2 vs. Fucci(SA)2 reporter systems in chicken DF1 fibroblasts. In the absence of serum, an increased proportion of cells are observed in G1 and S phase through DNA content analysis using flow cytometry. This shift is accurately captured in the fluorescent profiles of H1.0-Fucci(CA)2 but not H2B-Fucci(SA)2 cells. For comparison, control conditions are shown in Fig 2F and 2J. Schematics created in BioRender. Glover, J. (2026) https://BioRender.com/37cu02a.
(TIF)

S2 Fig Further characterisation of the viability and functionality of FuChi chickens. **A)** Table showing fertility, viability and transgene transmission rate of FuChi embryos across various developmental stages. **B)** FuChi chicks can be hatched, are viable, and reporter signal can be detected. **C)** Hatched FuChi chicks are smaller than wild-type chicks from the same clutch but gain weight at a similar rate. **D)** Mapped transgene integration sites in FuChi chickens based on analysis of a pool of five embryos. **E)** Percentage of chromosome integration sites identified by sequencing present in FuChi embryos. At least one additional transgene insertion is present that was not represented in the embryo pool analysed in (D). In total, 87 embryos were analysed at two developmental stages (HH19; *n* = 32; HH35; *n* = 55). **F)** Validation of H1.0-Fucci(CA)2 reporter system by flow analysis in two independent PGC lines derived from FuChi embryos in both control, growth factor-removed, and nocodazole (Nz)-treated conditions. The reporter profiles accurately reflect DNA content as determined by DAPI staining in both control and experimental conditions. Results from a third PGC line (#2) can be seen in Fig 3G. Schematics created in BioRender. Glover, J. (2026) https://BioRender.com/37cu02a. The underlying numerical data for this figure is detailed in S1 Data.
(TIF)

S3 Fig Parameterisation for image-based analysis of the H1.0-Fucci(CA)2 reporter in FuChi cells in vitro and in vivo. **A)** Representative confocal images of PGCs cultured under different conditions used for analysis of cell cycle stage. The same experimental cells that were analysed by flow cytometry (Figs 3G and S2F) were used. **B)** Scatter plots of relative mCherry and mVenus values from image-based analysis of FuChi PGCs. Gates for each cell cycle are marked. Pink, green and blue dots represent cells from three independently derived PGC lines. **C)** Quantification of cell cycle phase in PGCs by image-based and flow cytometry methodologies. For image-based quantification, >100 PGCs were analysed per line per condition. Nz = nocodazole. Scale bars = 50 µm. Error bars represent s.e.m. The underlying numerical data for this figure is detailed in S1 Data.
(TIF)

S4 Fig Reporter fluorescence quality post-fixation. **A)** Example images of FuChi or wild-type embryos which were fixed and/or stored in each medium condition as annotated and imaged at 0 and 72 hours to detect H1.0-mCerulean (mCerulean), mCherry-hCDT1 (mCherry) or mVenus-hGMNN (mVenus) fluorescence. **B)** Fluorescent reporter levels at 0 and 72 hours in FuChi and wild-type embryos following fixation and/or storage. Fixation in 10% neutral buffered formalin (NBF) or 4% paraformaldehyde (PFA) stabilises H1.0-Fucci(CA)2 reporter signal while reducing the increase in autofluorescence levels observed without fixation. Error bars represent S.E.M. *p < 0.05, **p < 0.01, ***p < 0.001; two-way repeated-measures ANOVAs followed by Tukey’s multiple comparisons tests. Scale bars = 500 µm. Error bars represent s.e.m. The underlying numerical data for this figure is detailed in S1 Data.
(TIF)

S5 Fig Detection of cell-cycle reporter profiles by antibody staining in paraffin-embedded tissues. **A)** Immunofluorescent staining of formalin-fixed paraffin-embedded (FFPE) brain sections detecting the His-tag, fused to mCherry-hCDT1, and the Myc tag, fused to mVenus-hGMNN. EDTA-based antigen retrieval enabled specific detection of both tags, revealing the proliferative profile of the brain tissue. Proliferative cells (green) were largely restricted to the neuroepithelium (Ne) and choroid plexus (Cp), consistent with native reporter signal observed in cryosections of FuChi embryos. **B)** Antibody staining of FFPE intestinal sections from FuChi embryos revealed a reporter profile similar to that observed in FuChi intestinal cryosections, with proliferative cells mainly localised to the epithelial layers. Asterisks indicate non-specific staining. **C)** Antibody staining of FFPE neural tube sections detected the characteristic proliferative profile of the neural tube: apical cells were predominantly in S/G2/M phase and appeared green, whereas basal cells were predominantly in G1 phase. **D)** Proteinase K treatment provided an alternative antigen retrieval approach for detecting His-tag expression in FFPE sections; however, co-staining with the Myc antibody used in panels A–C was not compatible due to loss of Myc signal with proteinase K treatment. **E)** Although not compatible with His-tag co-staining, a second primary antibody was identified for detection of the Myc tag in FFPE FuChi sections, producing strong staining across antigen retrieval conditions. Further details of staining conditions are provided in Table 2. Scale bars, 50 µm.
(TIF)

S6 Fig Cell cycle analysis in developing FuChi embryos using light sheet microscopy. **A)** Cell counts of selected embryonic and extraembryonic regions of the FuChi embryo visualised by light sheet microscopy across time in Fig 7C. All cells detected in the analysed regions were categorised based on their fluorescence profile at the time interval; mCherry-hCDT1-positive (G1; magenta), mVenus-hGMNN-positive (S; green), or positive in both channels (Late S, G2/M; black). These results are representative of four independent repeats. **B)** 3D epiblast (Ep) views of a developing FuChi embryo at two time points approximately 6 hours apart. Four optical sections (i–iv), taken from anterior (Ant.) to posterior (Pos.), are shown at the positions indicated by white lines. Note the difference in cell cycle status (G1) of cells egressing from the primitive streak (Ps), indicated by arrows. Scale bars as annotated. The underlying numerical data for this figure is detailed in S1 Data.
(TIF)

S1 Video Imaging of DF1 cells expressing the H1.0-Fucci(CA)2 biosensor. These cells express H1.0-mCerulean (cyan), mCherry-hCDT1(1/100)PIP(+)Cy(−) (magenta), and mVenus-hGMNN(1/110) (green) fusion proteins. Images were captured every 10 min by confocal microscopy. Scale bar = 50 µm.
(MP4)

S2 Video Imaging of DF1 cells expressing the H2B-Fucci(SA)2 biosensor. These cells express H2B-mCerulean (cyan), mCherry-hCDT1(30/120)PIP(−)Cy(+) (magenta), and mVenus-hGMNN(1/110) (green) fusion proteins. Images were captured every 10 min by confocal microscopy. Scale bar = 50 µm.
(MP4)

S3 Video Visualisation of H1.0-Fucci(CA)2 biosensor activity in limb micromass cell cultures derived from a FuChi embryo. mCherry-hCDT1 (magenta), mVenus-hGMNN (green), and H1.0-mCerulean (cyan) show dynamic fluorescent reporter activity during cell division.Images were captured every 10 min by confocal microscopy. Scale bar = 50 µm.
(MP4)

S4 Video Live imaging of a developing FuChi embryo from HH4. Left panel: mCherry-hCDT1 (magenta) and mVenus-hGMNN (green) signal. Hensen’s node (Hn) displays co-labelling of both reporters (G2/M), whereas the forming notochord (Nc) is enriched for mCherry-hCDT1 labelling (G1). Right panel: H1.0-mCerulean (cyan) reporter signal. Images were captured every 15 min using an Axio Zoom.V16 stereo zoom microscope. Scale bar = 500 µm.
(MP4)

S5 Video Time-lapse imaging of head fold formation in a FuChi embryo. Left panel: mCherry-hCDT1 (magenta) and mVenus-hGMNN (green) signal. Right panel: H1.0-mCerulean (cyan) reporter signal. Images were captured every 10 min by confocal microscopy. Scale bar = 100 µm.
(MP4)

S6 Video Visualisation of somite formation in FuChi embryos. The initial part of the video shows the sequential formation of somites in a FuChi embryo; left panel: mCherry-hCDT1 (magenta) and mVenus-hGMNN (green) signal; right panel: H1.0-mCerulean (cyan) reporter signal. Scale bar = 100 µm. The second part of the video shows magnified views of individual somites visualised using the mCherry-hCDT1 and mVenus-hGMNN reporters. Images were captured every 15 min by confocal microscopy. Scale bar = 15 µm.
(MP4)

S7 Video Light sheet imaging of a developing FuChi embryo. Changes in cell cycle dynamics in the epiblast during development from EG and K stage XII to HH4. Time interval between images is 8 min. Scale bar = 500 µm.
(MP4)

S8 Video The dynamic position of selected 330 x 330 µm regions in the embryonic and extraembryonic areas selected for analysis. The regions (yellow squares) were tracked over time using the velocity field calculated using particle image velocimetry. The time interval between images is 10 min. Scale bar = 500 µm.
(MP4)

S9 Video Cutout of a small epiblast region from the embryonic area of a developing FuChi embryo. Coloured circles indicate detection of mVenus-hGMNN (green) or mCherry-hCDT1 (magenta) fluorescence. Manually tracked cells are outlined by blue squares. Scale bar = 50 µm.
(MP4)

S10 Video Cutout of a small epiblast region from the extraembryonic tissue of a developing FuChi embryo. Coloured circles indicate detection of mVenus-hGMNN (green) or mCherry-hCDT1 (magenta) fluorescence. Manually tracked cells are outlined by blue squares. Scale bar = 50 µm.
(MP4)

S11 Video Hypoblast side-up view of the hypoblast and emerging mesendoderm in a developing FuChi embryo. Light sheet imaging of a FuChi embryo developing from HH3 to HH5, with a depth of view of 60 µm. The time interval between images is 10 min. Scale bar = 500 µm.
(MP4)

S1 Data Raw data used for the quantification and analyses within this paper. The relevant figure is indicated in the title of each spreadsheet.
(XLSX)

## Abbreviations

APC^Cdh1^: Cdh1-activated anaphase-promoting complex ubiquitin ligase complex
CDT1: chromatin licensing and DNA replication factor 1
CUL4^Ddb1^: Cullin 4-Ddb1 ubiquitin ligase complex
Cy motif: Cyclin-binding motif
EG and K: Eyal-Giladi and Kochav
FFPE: formalin-fixed paraffin-embedded
Fucci: fluorescent ubiquitination-based cell cycle indicator
FuChi: Fucci Chicken
GMNN: geminin
HCR: hybridisation chain reaction
HH: Hamburger–Hamilton
PGC: primordial germ cell
PIP box: PCNA-interacting protein box
PSM: presomitic mesoderm
SCF^Skp2^: Skp2-containing Skp1-Cullin-F-box ubiquitin ligase complex
YAP1: Yes-associated protein 1

## References

[1] Edgar B. Diversification of cell cycle controls in developing embryos. Curr Opin Cell Biol. 1995;7(6):815–24. doi: 10.1016/0955-0674(95)80065-4 8608012

[2] Nogawa H, Morita K, Cardoso WV. Bud formation precedes the appearance of differential cell proliferation during branching morphogenesis of mouse lung epithelium in vitro. Dev Dyn. 1998;213(2):228–35. doi: 10.1002/(SICI)1097-0177(199810)213:2<228::AID-AJA8>3.0.CO;2-I 9786423

[3] Michael L, Davies JA. Pattern and regulation of cell proliferation during murine ureteric bud development. J Anat. 2004;204(4):241–55. doi: 10.1111/j.0021-8782.2004.00285.x 15061751 PMC1571296

[4] Pickering J, Chinnaiya K, Towers M. An autoregulatory cell cycle timer integrates growth and specification in chick wing digit development. Elife. 2019;8:e47625. doi: 10.7554/eLife.47625 31545166 PMC6777937

[5] Nishitani H, Sugimoto N, Roukos V, Nakanishi Y, Saijo M, Obuse C, et al. Two E3 ubiquitin ligases, SCF-Skp2 and DDB1-Cul4, target human Cdt1 for proteolysis. EMBO J. 2006;25(5):1126–36. doi: 10.1038/sj.emboj.7601002 16482215 PMC1409712

[6] Vodermaier HC. APC/C and SCF: Controlling each other and the cell cycle. Curr Biol. 2004;14(18):R787-96. doi: 10.1016/j.cub.2004.09.020 15380093

[7] Wei W, Ayad NG, Wan Y, Zhang G-J, Kirschner MW, Kaelin WG Jr. Degradation of the SCF component Skp2 in cell-cycle phase G1 by the anaphase-promoting complex. Nature. 2004;428(6979):194–8. doi: 10.1038/nature02381 15014503

[8] Benmaamar R, Pagano M. Involvement of the SCF complex in the control of Cdh1 degradation in S-phase. Cell Cycle. 2005;4(9):1230–2. doi: 10.4161/cc.4.9.2048 16123585

[9] Nishitani H, Lygerou Z, Nishimoto T. Proteolysis of DNA replication licensing factor Cdt1 in S-phase is performed independently of geminin through its N-terminal region. J Biol Chem. 2004;279(29):30807–16. doi: 10.1074/jbc.M312644200 15138268

[10] Ang XL, Harper JW. Interwoven ubiquitination oscillators and control of cell cycle transitions. Sci STKE. 2004;2004(242). doi: 10.1126/stke.2422004pe31 15266102

[11] Sakaue-Sawano A, Yo M, Komatsu N, Hiratsuka T, Kogure T, Hoshida T, et al. Genetically encoded tools for optical dissection of the mammalian cell cycle. Mol Cell. 2017;68(3):626-640.e5. doi: 10.1016/j.molcel.2017.10.001 29107535

[12] Mort RL, Ford MJ, Sakaue-Sawano A, Lindstrom NO, Casadio A, Douglas AT, et al. Fucci2a: a bicistronic cell cycle reporter that allows Cre mediated tissue specific expression in mice. Cell Cycle. 2014;13(17):2681–96. doi: 10.4161/15384101.2015.945381 25486356 PMC4613862

[13] Sakaue-Sawano A, Kurokawa H, Morimura T, Hanyu A, Hama H, Osawa H, et al. Visualizing spatiotemporal dynamics of multicellular cell-cycle progression. Cell. 2008;132(3):487–98. doi: 10.1016/j.cell.2007.12.033 18267078

[14] Sakaue-Sawano A, Kobayashi T, Ohtawa K, Miyawaki A. Drug-induced cell cycle modulation leading to cell-cycle arrest, nuclear mis-segregation, or endoreplication. BMC Cell Biol. 2011;12:2. doi: 10.1186/1471-2121-12-2 21226962 PMC3277280

[15] Ford MJ, Yeyati PL, Mali GR, Keighren MA, Waddell SH, Mjoseng HK, et al. A cell/cilia cycle biosensor for single-cell kinetics reveals persistence of cilia after G1/S transition is a general property in cells and mice. Dev Cell. 2018;47(4):509-523.e5. doi: 10.1016/j.devcel.2018.10.027 30458140 PMC6251972

[16] Ando R, Sakaue-Sawano A, Shoda K, Miyawaki A. Two coral fluorescent proteins of distinct colors for sharp visualization of cell-cycle progression. Cell Struct Funct. 2023;48(2):135–44. doi: 10.1247/csf.23028 37394513 PMC10958192

[17] Carlier G, Maugein A, Cordier C, Pechberty S, Garfa-Traoré M, Martin P, et al. Human fucci pancreatic Beta cell lines: New tools to study Beta cell cycle and terminal differentiation. PLoS One. 2014;9(9):e108202. doi: 10.1371/journal.pone.0108202 25259951 PMC4178124

[18] Sugiyama M, Sakaue-Sawano A, Iimura T, Fukami K, Kitaguchi T, Kawakami K, et al. Illuminating cell-cycle progression in the developing zebrafish embryo. Proc Natl Acad Sci U S A. 2009;106(49):20812–7. doi: 10.1073/pnas.0906464106 19923430 PMC2779202

[19] Cura Costa E, Otsuki L, Rodrigo Albors A, Tanaka EM, Chara O. Spatiotemporal control of cell cycle acceleration during axolotl spinal cord regeneration. Elife. 2021;10:e55665. doi: 10.7554/eLife.55665 33988504 PMC8205487

[20] Bajar BT, Lam AJ, Badiee RK, Oh Y-H, Chu J, Zhou XX, et al. Fluorescent indicators for simultaneous reporting of all four cell cycle phases. Nat Methods. 2016;13(12):993–6. doi: 10.1038/nmeth.4045 27798610 PMC5548384

[21] Davey MG, Tickle C. The chicken as a model for embryonic development. Cytogenet Genome Res. 2007;117(1–4):231–9. doi: 10.1159/000103184 17675864

[22] Mok GF, Alrefaei AF, McColl J, Grocott T, Münsterberg A. Chicken as a developmental model. Encyclopedia of Life Sciences. Wiley; 2015. p. 1–8. doi: 10.1002/9780470015902.a0021543

[23] Esteves de Lima J, Bonnin M-A, Bourgeois A, Parisi A, Le Grand F, Duprez D. Specific pattern of cell cycle during limb fetal myogenesis. Dev Biol. 2014;392(2):308–23. doi: 10.1016/j.ydbio.2014.05.015 24882711

[24] Ecker A, Lázár B, Tóth RI, Urbán M, Hoffmann OI, Fekete Z, et al. Creating a novel method for chicken primordial germ cell health monitoring using the fluorescent ubiquitination-based cell cycle indicator reporter system. Poult Sci. 2024;103(10):104144. doi: 10.1016/j.psj.2024.104144 39173570 PMC11382113

[25] Loftus AEP, Romano MS, Phuong AN, McKinnel BJ, Muir MT, Furqan M, et al. An ILK/STAT3 pathway controls glioblastoma stem cell plasticity. Dev Cell. 2024;59(24):3197-3212.e7. doi: 10.1016/j.devcel.2024.09.003 39326421

[26] Yusa K, Zhou L, Li MA, Bradley A, Craig NL. A hyperactive piggyBac transposase for mammalian applications. Proc Natl Acad Sci U S A. 2011;108(4):1531–6. doi: 10.1073/pnas.1008322108 21205896 PMC3029773

[27] Whyte J, Glover JD, Woodcock M, Brzeszczynska J, Taylor L, Sherman A, et al. FGF, insulin, and SMAD signaling cooperate for avian primordial germ cell self-renewal. Stem Cell Reports. 2015;5(6):1171–82. doi: 10.1016/j.stemcr.2015.10.008 26677769 PMC4682126

[28] Hamburger V, Hamilton HL. A series of normal stages in the development of the chick embryo. J Morphol. 1951;88(1):49–92. doi: 10.1002/jmor.1050880104 24539719

[29] Ballantyne M, Woodcock M, Doddamani D, Hu T, Taylor L, Hawken RJ, et al. Direct allele introgression into pure chicken breeds using Sire Dam Surrogate (SDS) mating. Nat Commun. 2021;12(1):659. doi: 10.1038/s41467-020-20812-x 33510156 PMC7844028

[30] Schindelin J, Arganda-Carreras I, Frise E, Kaynig V, Longair M, Pietzsch T, et al. Fiji: An open-source platform for biological-image analysis. Nat Methods. 2012;9(7):676–82. doi: 10.1038/nmeth.2019 22743772 PMC3855844

[31] Van Kerckvoorde M, Ford MJ, Yeyati PL, Mill P, Mort RL. Live imaging and analysis of cilia and cell cycle dynamics with the Arl13bCerulean-Fucci2a biosensor and fucci tools. Methods Mol Biol. 2021;2329:291–309. doi: 10.1007/978-1-0716-1538-6_21 34085231

[32] Rozbicki E, Chuai M, Karjalainen AI, Song F, Sang HM, Martin R, et al. Myosin-II-mediated cell shape changes and cell intercalation contribute to primitive streak formation. Nat Cell Biol. 2015;17(4):397–408. doi: 10.1038/ncb3138 25812521 PMC4886837

[33] Chuai M, Serrano Nájera G, Serra M, Mahadevan L, Weijer CJ. Reconstruction of distinct vertebrate gastrulation modes via modulation of key cell behaviors in the chick embryo. Sci Adv. 2023;9(1):eabn5429. doi: 10.1126/sciadv.abn5429 36598979 PMC9812380

[34] Serra M, Streichan S, Chuai M, Weijer CJ, Mahadevan L. Dynamic morphoskeletons in development. Proc Natl Acad Sci U S A. 2020;117(21):11444–9. doi: 10.1073/pnas.1908803117 32381735 PMC7260943

[35] Oh JDH, Freem L, Saunders DDZ, McTeir L, Gilhooley H, Jackson M, et al. Insights into digit evolution from a fate map study of the forearm using Chameleon, a new transgenic chicken line. Development. 2024;151(13):dev202340. doi: 10.1242/dev.202340 38828852 PMC11234372

[36] Li H. New strategies to improve minimap2 alignment accuracy. Bioinformatics. 2021;37(23):4572–4. doi: 10.1093/bioinformatics/btab705 34623391 PMC8652018

[37] Li H, Handsaker B, Wysoker A, Fennell T, Ruan J, Homer N, et al. The Sequence Alignment/Map format and SAMtools. Bioinformatics. 2009;25:2078–9. doi: 10.1093/bioinformatics/btp352 PMC2723002 19505943

[38] Smith J, Alfieri JM, Anthony N, Arensburger P, Athrey GN, Balacco J, et al. Fourth report on chicken genes and chromosomes 2022. Cytogenet Genome Res. 2022;162:405–528. doi: 10.1159/000529376 PMC11835228 36716736

[39] Choi HMT, Schwarzkopf M, Fornace ME, Acharya A, Artavanis G, Stegmaier J, et al. Third-generation in situ hybridization chain reaction: multiplexed, quantitative, sensitive, versatile, robust. Development. 2018;145(12):dev165753. 10.1242/dev.165753 PMC6031405 29945988

[40] Chapman SC, Collignon J, Schoenwolf GC, Lumsden A. Improved method for chick whole-embryo culture using a filter paper carrier. Dev Dyn. 2001;220(3):284–9. doi: 10.1002/1097-0177(20010301)220:3<284::AID-DVDY1102>3.0.CO;2-5 11241836

[41] Pauklin S, Vallier L. The cell-cycle state of stem cells determines cell fate propensity. Cell. 2013;155(1):135–47. doi: 10.1016/j.cell.2013.08.031 24074866 PMC3898746

[42] Verma S, Moreno IY, Gesteira TF, Coulson-Thomas VJ. Toxicity of nuclear-localized GFP in reporter mice. Sci Rep. 2024;14(1):24642. doi: 10.1038/s41598-024-75741-2 39428407 PMC11491490

[43] Ding S, Wu X, Li G, Han M, Zhuang Y, Xu T. Efficient transposition of the piggyBac (PB) transposon in mammalian cells and mice. Cell. 2005;122(3):473–83. doi: 10.1016/j.cell.2005.07.013 16096065

[44] Pokhrel N, Genin O, Sela-Donenfeld D, Cinnamon Y. HREM, RNAseq and cell cycle analyses reveal the role of the G2/M-Regulatory protein, WEE1, on the survivability of chicken embryos during diapause. Biomedicines. 2022;10(4):779. doi: 10.3390/biomedicines10040779 35453529 PMC9033001

[45] Sanders EJ, Varedi M, French AS. Cell proliferation in the gastrulating chick embryo: A study using BrdU incorporation and PCNA localization. Development. 1993;118(2):389–99. doi: 10.1242/dev.118.2.389 7900990

[46] Solovieva T, Lu H-C, Moverley A, Plachta N, Stern CD. The embryonic node behaves as an instructive stem cell niche for axial elongation. Proc Natl Acad Sci U S A. 2022;119(5):e2108935119. doi: 10.1073/pnas.2108935119 35101917 PMC8812687

[47] Abe T, Sakaue-Sawano A, Kiyonari H, Shioi G, Inoue K, Horiuchi T, et al. Visualization of cell cycle in mouse embryos with Fucci2 reporter directed by Rosa26 promoter. Development. 2013;140(1):237–46. doi: 10.1242/dev.084111 23175634

[48] Kasioulis I, Storey KG. Cell biological mechanisms regulating chick neurogenesis. Int J Dev Biol. 2018;62(1-2–3):167–75. doi: 10.1387/ijdb.170268ks 29616725

[49] Das RM, Storey KG. Mitotic spindle orientation can direct cell fate and bias Notch activity in chick neural tube. EMBO Rep. 2012;13(5):448–54. doi: 10.1038/embor.2012.42 22491029 PMC3343353

[50] Wilcock AC, Swedlow JR, Storey KG. Mitotic spindle orientation distinguishes stem cell and terminal modes of neuron production in the early spinal cord. Development. 2007;134(10):1943–54. doi: 10.1242/dev.002519 17470968 PMC7116174

[51] Modak SP, Morris G, Yamada T. DNA synthesis and mitotic activity during early development of chick lens. Dev Biol. 1968;17(5):544–61. doi: 10.1016/0012-1606(68)90004-3 5658458

[52] Rapaport DH, Wong LL, Wood ED, Yasumura D, LaVail MM. Timing and topography of cell genesis in the rat retina. J Comp Neurol. 2004;474(2):304–24. doi: 10.1002/cne.20134 15164429

[53] Silva AO, Ercole CE, McLoon SC. Plane of cell cleavage and numb distribution during cell division relative to cell differentiation in the developing retina. J Neurosci. 2002;22(17):7518–25. doi: 10.1523/JNEUROSCI.22-17-07518.2002 12196575 PMC6757979

[54] Gupta S, Maurya R, Saxena M, Sen J. Defining structural homology between the mammalian and avian hippocampus through conserved gene expression patterns observed in the chick embryo. Dev Biol. 2012;366(2):125–41. doi: 10.1016/j.ydbio.2012.03.027 22537492

[55] Shyer AE, Tallinen T, Nerurkar NL, Wei Z, Gil ES, Kaplan DL, et al. Villification: How the gut gets its villi. Science. 2013;342(6155):212–8. doi: 10.1126/science.1238842 23989955 PMC4045245

[56] Walton KD, Mishkind D, Riddle MR, Tabin CJ, Gumucio DL. Blueprint for an intestinal villus: Species-specific assembly required. Wiley Interdiscip Rev Dev Biol. 2018;7(4):e317. doi: 10.1002/wdev.317 29513926 PMC6002883

[57] Arsenault P, Ménard D. Cell proliferation in developing human jejunum. Biol Neonate. 1987;51(6):297–304. doi: 10.1159/000242668 3607106

[58] Gill HK, Yin S, Lawlor JC, Huycke TR, Nerurkar NL, Tabin CJ, et al. The developmental mechanics of divergent buckling patterns in the chick gut. Proc Natl Acad Sci U S A. 2024;121(28):e2310992121. doi: 10.1073/pnas.2310992121 38968105 PMC11252809

[59] Pearse RV 2nd, Vogan KJ, Tabin CJ. Ptc1 and Ptc2 transcripts provide distinct readouts of Hedgehog signaling activity during chick embryogenesis. Dev Biol. 2001;239(1):15–29. doi: 10.1006/dbio.2001.0430 11784016

[60] Aglyamova GV, Agarwala S. Gene expression analysis of the hedgehog signaling cascade in the chick midbrain and spinal cord. Dev Dyn. 2007;236(5):1363–73. doi: 10.1002/dvdy.21146 17436280

[61] Seo S, Kim YA, Lee J, Lee S, Kim J, Lee S. Fat3 regulates neural progenitor cells by promoting Yap activity during spinal cord development. Sci Rep. 2022;12(1):14726. doi: 10.1038/s41598-022-19029-3 36042367 PMC9427758

[62] Morimoto M, Saito D. Avian primordial germ cell migration: History, mechanisms, applications, and unanswered questions. Protein Expr Purif. 2025;235:106788. doi: 10.1016/j.pep.2025.106788 40752581

[63] Nakamura Y, Yamamoto Y, Usui F, Mushika T, Ono T, Setioko AR, et al. Migration and proliferation of primordial germ cells in the early chicken embryo. Poult Sci. 2007;86(10):2182–93. doi: 10.1093/ps/86.10.2182 17878448

[64] Yang SY, Lee HJ, Lee HC, Hwang YS, Park YH, Ono T, et al. The dynamic development of germ cells during chicken embryogenesis. Poult Sci. 2018;97(2):650–7. doi: 10.3382/ps/pex316 29126291

[65] Eyal-Giladi H, Kochav S. From cleavage to primitive streak formation: A complementary normal table and a new look at the first stages of the development of the chick. I. General morphology. Dev Biol. 1976;49(2):321–37. doi: 10.1016/0012-1606(76)90178-0 944662

[66] Takeda DY, Parvin JD, Dutta A. Degradation of Cdt1 during S phase is Skp2-independent and is required for efficient progression of mammalian cells through S phase. J Biol Chem. 2005;280(24):23416–23. doi: 10.1074/jbc.M501208200 15855168

[67] Liu E, Li X, Yan F, Zhao Q, Wu X. Cyclin-dependent kinases phosphorylate human Cdt1 and induce its degradation. J Biol Chem. 2004;279:17283–8. doi: 10.1074/jbc.C300549200 15004027

[68] Arias EE, Walter JC. Strength in numbers: Preventing rereplication via multiple mechanisms in eukaryotic cells. Genes Dev. 2007;21(5):497–518. doi: 10.1101/gad.1508907 17344412

[69] Chen D, Zhi Y, Jia X, Ji N, Zhang J, Liang J, et al. Characterization of primordial germ cells from EG&K stage X chicken embryos. Poult Sci. 2025;104(8):105308. doi: 10.1016/j.psj.2025.105308 40451075 PMC12163151

[70] Rengaraj D, Cha DG, Park KJ, Lee KY, Woo SJ, Han JY. Finer resolution analysis of transcriptional programming during the active migration of chicken primordial germ cells. Comput Struct Biotechnol J. 2022;20:5911–24. doi: 10.1016/j.csbj.2022.10.034 36382185 PMC9636429

[71] Kang KS, Lee HC, Kim HJ, Lee HG, Kim YM, Lee HJ, et al. Spatial and temporal action of chicken primordial germ cells during initial migration. Reproduction. 2015;149(2):179–87. doi: 10.1530/REP-14-0433 25550524

[72] Khudyakov J, Bronner-Fraser M. Comprehensive spatiotemporal analysis of early chick neural crest network genes. Dev Dyn. 2009;238(3):716–23. doi: 10.1002/dvdy.21881 19235729 PMC2650819

[73] Selleck MA, Bronner-Fraser M. The genesis of avian neural crest cells: A classic embryonic induction. Proc Natl Acad Sci U S A. 1996;93(18):9352–7. doi: 10.1073/pnas.93.18.9352 8790333 PMC38431

[74] Stern CD, Bellairs R. Mitotic activity during somite segmentation in the early chick embryo. Anat Embryol (Berl). 1984;169(1):97–102. doi: 10.1007/BF00300591 6721226

[75] Collier JR, Mcinerney D, Schnell S, Maini PK, Gavaghan DJ, Houston P, et al. A cell cycle model for somitogenesis: Mathematical formulation and numerical simulation. J Theor Biol. 2000;207(3):305–16. doi: 10.1006/jtbi.2000.2172 11082301

[76] Bulusu V, Prior N, Snaebjornsson MT, Kuehne A, Sonnen KF, Kress J, et al. Spatiotemporal analysis of a glycolytic activity gradient linked to mouse embryo mesoderm development. Dev Cell. 2017;40(4):331-341.e4. doi: 10.1016/j.devcel.2017.01.015 28245920 PMC5337618

[77] Horikawa K, Ishimatsu K, Yoshimoto E, Kondo S, Takeda H. Noise-resistant and synchronized oscillation of the segmentation clock. Nature. 2006;441(7094):719–23. doi: 10.1038/nature04861 16760970

[78] van Oostrom MJ, Li YI, Meijer WHM, Noordzij TEJC, Fountas C, Timmers E, et al. Coupling of cell proliferation to the segmentation clock ensures robust somite scaling. bioRxiv. 2025. doi: 10.1101/2025.01.10.632257

[79] Mort RL, Keighren M, Hay L, Jackson IJ. Ex vivo culture of mouse embryonic skin and live-imaging of melanoblast migration. J Vis Exp. 2014;(87):51352. doi: 10.3791/51352 24894489 PMC4193345

[80] Stern CD. The chick; a great model system becomes even greater. Dev Cell. 2005;8(1):9–17. doi: 10.1016/j.devcel.2004.11.018 15621526

[81] van de Lavoir M-C, Diamond JH, Leighton PA, Mather-Love C, Heyer BS, Bradshaw R, et al. Germline transmission of genetically modified primordial germ cells. Nature. 2006;441(7094):766–9. doi: 10.1038/nature04831 16760981

[82] Macdonald J, Taylor L, Sherman A, Kawakami K, Takahashi Y, Sang HM, et al. Efficient genetic modification and germ-line transmission of primordial germ cells using piggyBac and Tol2 transposons. Proc Natl Acad Sci U S A. 2012;109(23):E1466-72. doi: 10.1073/pnas.1118715109 22586100 PMC3384192

[83] Doddamani D, Carlson DF, McTeir L, Taylor L, Nandi S, Davey MG, et al. PRDM14 is essential for vertebrate gastrulation and safeguards avian germ cell identity. Dev Biol. 2025;521:129–37. doi: 10.1016/j.ydbio.2025.02.005 39938772

[84] Ho Ching Chan B, Hardy H, Requena T, Findlay A, Ioannidis J, Meunier D, et al. A stable NTN1 fluorescent reporter chicken reveals cell specific molecular signatures during optic fissure closure. Sci Rep. 2025;15(1):10096. doi: 10.1038/s41598-025-94589-8 40128351 PMC11933247

[85] Idoko-Akoh A, Goldhill DH, Sheppard CM, Bialy D, Quantrill JL, Sukhova K, et al. Creating resistance to avian influenza infection through genome editing of the ANP32 gene family. Nat Commun. 2023;14(1):6136. doi: 10.1038/s41467-023-41476-3 37816720 PMC10564915

[86] McGrew MJ, Sherman A, Lillico SG, Ellard FM, Radcliffe PA, Gilhooley HJ, et al. Localised axial progenitor cell populations in the avian tail bud are not committed to a posterior Hox identity. Development. 2008;135(13):2289–99. doi: 10.1242/dev.022020 18508860

[87] Hagihara Y, Okuzaki Y, Matsubayashi K, Kaneoka H, Suzuki T, Iijima S, et al. Primordial germ cell-specific expression of eGFP in transgenic chickens. Genesis. 2020;58(8):e23388. doi: 10.1002/dvg.23388 32776392

[88] Wang Y, Xue W, Pustovalova M, Kuzmin DV, Leonov S. Chick embryo chorioallantoic membrane (CAM) model for cancer studies and drug evaluation. Front Biosci (Landmark Ed). 2025;30. doi: 10.31083/fbl37456 40464506

[89] McGrew MJ, Holmes T, Davey MG. A scientific case for revisiting the embryonic chicken model in biomedical research. Dev Biol. 2025;522:220–6. doi: 10.1016/j.ydbio.2025.02.013 40015500

[90] Archit A, Freckmann L, Nair S, Khalid N, Hilt P, Rajashekar V, et al. Segment anything for microscopy. Nat Methods. 2025;22(3):579–91. doi: 10.1038/s41592-024-02580-4 39939717 PMC11903314

[91] Bragantini J, Theodoro I, Zhao X, Huijben TAPM, Hirata-Miyasaki E, VijayKumar S, et al. Ultrack: Pushing the limits of cell tracking across biological scales. Nat Methods. 2025;22(11):2423–36. doi: 10.1038/s41592-025-02778-0 40855368 PMC12615266

