# Supplementary material for: FuChi is a cell cycle biosensor for tracking cell cycle dynamics during avian development
Source: PLoS Biol. 2026 Oct 9;24(10):e3004036. doi: 10.1371/journal.pbio.3004036 (PMC13705591; doi:10.1371/journal.pbio.3004036)
Supplement: Supplementary file 2 [file pbio.3004036.s020.docx]

**Manuscript number:** RC-2025-03259

**Corresponding author(s):** James, Glover

*[The “revision plan” should delineate the revisions that authors intend to carry out in response to the points raised by the referees. It also provides the authors with the opportunity to explain their view of the paper and of the referee reports.*

*The document is important for the editors of affiliate journals when they make a first decision on the transferred manuscript. It will also be useful to readers of the reprint and help them to obtain a balanced view of the paper.*

*If you wish to submit a full revision, please use our "*[*Full Revision*](https://reviewcommons.org/templates/full-revision.docx)*" template.* ***It is important to use the appropriate template to clearly inform the editors of your intentions.****]*

## 1. General Statements [optional]

*This section is optional. Insert here any general statements you wish to make about the goal of the study or about the reviews.*

The main goals of this study are to introduce FuChi (Fucci Chicken), the first avian cell cycle reporter model, and to demonstrate the superior nature of our tricistronic Fucci(CA)2 biosensor at delineating all four cell cycle stages *in vivo* compared to previous *in vivo* models (including mouse). The additional utility of our construct provides compatibility with downstream applications including *in situ* hybridisation and immunofluorescence (including on paraffin embedded sections), making this an invaluable new resource. Furthermore, we have also provided novel discoveries as proof of concept using our reporter embryos, including the cell cycle dynamics that cells undergo during vertebrate gastrulation, and how early germ cells migrate. Our work not only introduces a powerful new cell cycle biosensor expressing chicken line but also positions this line as the best available *in vivo* model for studying cell cycle dynamics.

We would like to thank all the reviewers for taking the time and effort to thoroughly assess our manuscript and provide very useful comments and suggestions. Overall, the reviewers were very positive and all agreed that it was a major advance, and would be of broad interest and utility to the biological community. We agree with the reviewers that the readability and accessibility of the paper could be improved, and we have addressed this in our revision to ensure clarity for a wider audience.

## 2. Description of the planned revisions

*Insert here a point-by-point reply that explains what revisions, additional experimentations and analyses are planned to address the points raised by the referees.*

Please find below a point-by-point reply to address points raised by the referees, where revisions, experiments or analyses are planned.

**Reviewer #1**

- With regards to the reviewer’s comment regarding the difficulty in drawing conclusions ‘about cell cycle in different tissue layers without sectioning’ in Figure 3 we will include more sections of FuChi embryos which include structures such as mesenchymal condensates.
- To address the clarity regarding ‘cell cycle status changes as cells egress form the primitive streak, to form prechordal plate’ we will add sections of gastrulating FuChi embryos to further demonstrate the cell cycle status of cells that form the pre-chordal plate.

**Reviewer #2**

Major comments:

Reviewer 2 made very valuable observations and suggestions regarding our data and interpretation of somitogenesis, specifically in response to our sentence saying that “the mesenchyme, which is predominantly in G1 as they undergo condensation”. Furthermore, they noted that Supplementary Video 4 “shows distinct green fluorescence (S) in the presomitic mesoderm for the first hour or so, only then turning to magenta (G1)”. We were asked to review the sentence/video to clarify if this is a significant finding or if this is not representative of their observations.

- We thank the reviewer for this suggestion. From looking again at our timelapse movies, and also analysing additional static images, we agree that presomitic mesoderm (PSM) does appear to be green (S phase), which then may transition to G1 as the somites form. To address this, we plan to quantify cell cycle status in the PSM on embryos to see if this is a significant finding.
- We hope this quantification of the PSM may also enable us to include discussion on how our findings relate to the Cell Cycle model for somitogenesis proposed in the Collier et al, 2000 paper suggested by the Reviewer.
- We agree with the Reviewer that “the fluorescence profiles in Figure 4C do not seem similar regarding the Myc-tag epitope” and believe this difference is likely just a reflection of the part of the image we used. We will include a more representative image once we have repeated the staining.
- Reviewer 2 has asked for quantitative support for our fluorescence-based interpretations. We thank the reviewer for this suggestion and are now planning to perform quantitative analyses of different tissues (similar to our quantification in germ cells; Figure 5F in revised manuscript) and in embryos to support our observations. These will include the PSM (see above), neural tube, intestine, and early embryos (also see Section 3 for blastoderm quantification).

**Reviewer #3**

Minor comments

- To enhance speedy interpretation, we will process our supplementary videos to include annotations and arrows to highlight key cells and events (e.g., a cell undergoing mitosis).
- As recommended by Reviewer 3, we have now quantified cell cycle status in blastoderm cells (see graph below), confirming that a high proportion are in the G2/M phase. We will include these data in the final revision, which will complement our planned quantification of cell cycle status in other tissues (see response to Reviewer 2).

- For our final revision, we will include higher magnification/zoomed in images of selected regions of the somites, neural tube (lumen) and retina (epithelium). Revisiting our images of the neural tube showed that dividing cells in the lumen did so in the perpendicular plane and we will include these images in our revision to provide further evidence of the fidelity of the FuChi reporter. We thank the reviewer for this excellent idea to show the efficacy of our system.
- To address the levels of proliferation in somites, we plan to generate a cropped video with a fixed ROI to enable proliferation in individual cells of the forming somites to be more readily visualised. This will be further complemented by the quantification of cell cycle status in forming somites (see responses to other reviewers).

## 3. Description of the revisions that have already been incorporated in the transferred manuscript

*Please insert a point-by-point reply describing the revisions that were already carried out and included in the transferred manuscript. If no revisions have been carried out yet, please leave this section empty.*

Please find below a point-by-point reply to address the points raised by the referees where we have already undertaken revisions or performed additional experiments/analyses that are included in this initial revision.

**Reviewer #1**

Major comments:

Reviewer 1 commented that the 'manuscript would greatly benefit from having someone spend time on the figures, and associated text, to ensure they are fully comprehensible’. We agree wholeheartedly with the reviewer and apologise. We have now revisited the text, figures, and associated figure legends to ensure that they are more easily accessible and fully comprehensible to readers from across disciplines. This includes adding labels to point out specific anatomical features on images. Further specific examples are outlined in the points below.

- In response to concerns raised by Reviewer 1 relating to: Figure 1 and the lack of figure citations; 'the persistence of mCherry in the H2B Fucci'; how mCherry seems to persist longer in H1 (compare Figs 1D and 1G)’:
  - We apologise for the lack of figure citation in the text. We have now reworked the figures relating to the constructs (original Figures 1 and S1) and have made these Figures 1, 2, and S1 in our updated version.
  - Figure 1 is now an introductory background figure which illustrates the differences between Fucci(SA) and Fucci(CA) reporters, with additional details provided in the associated legend, and call outs to the figure starting in the introduction.
  - Regarding ‘the persistence of mCherry in the H2B Fucci’, what we are trying to articulate is that the mCherry degradation that we observed in the H2B-Fucci(SA)2 expressing DF1 cells extended beyond the end of S phase and into G2/M, compared with what would be expected (revised Figure 2H, arrows).
  - We have now replaced these montages with a more representative example. Additionally, the new images (Figures 2C and 2G in revised manuscript) are synchronised (both starting at G2/M), restricted to a single cell cycle, are larger in size, and have the cell cycle stage labelled. We believe these changes will aid interpretation.
- Specifically relating to the lack of labelling in Figure 3A, we agree that this figure was not labelled sufficiently, and neither was there enough detail included in the text or figure legend for readers to follow easily and make their own conclusions. We have now added additional labels to this figure, broken the figure down into more panels (Figures 4A-4D in revised manuscript), and included more detailed descriptions in the associated figure legend and text.
- We thank the reviewer for making the important point that it is ‘hard to know where the biosensor is reporting patterns that are already well established (eg neural tube), and where the biosensor is reporting patterns that are novel - and if so, what these patterns are’ which was made more challenging by insufficient references to previous studies.
  - Firstly, as for the point above, we have now added labels to many of the panels (Figure 4 in revision), including highlighting features such as the non-proliferative dermal condensates and demarcating the proliferative retinal pigmented epithelium (Figures 4F and 4G in revision). Secondly, we have also now included additional references in the text where our proliferation profiles are consistent with previous literature, such as in the neural tube.
- To make our data on cell cycle stages as ‘cells egress from the primitive streak, to form prechordal plate’ clearer we have added additional labels to the figures (Figures 4A and 6E in revised manuscript) and this will be complemented by the planned embryo sectioning relating to this point (see Section 2).

Minor comments

- We have added additional references relating to the data in original Figure 3 (now Figure 4, see above), and any new descriptions of known proliferation profiles that we include will have appropriate citations.
- In this current revision we have addressed figure call out issues, and added labels to enhance readability, clarity and data interpretation.
- We have labelled figures more carefully and ensured figures and text align. See above.

**Reviewer #2**

Major comments

- Reviewer 2 rightly pointed out that the ‘description of the bicistronic tandem-Fucci(CA) system in paragraph 6 is not consistent with what is described in the original bibliographic reference indicated by the authors’. We have now added additional text to properly explain the CDT1 probe dynamics, as per the cited manuscript, and also referenced the schematics to help readers.
- To address whether the FuChi model can be accurately ‘used to study embryogenesis’ and following up on the suggestion to ‘indicate if the size of the embryos is comparable to the wildtype’ we have now included size comparisons of FuChi and wild-type/non-transgenic embryos at mid (E9) and late (E18) gestational stages demonstrating that there is no significant difference between genotypes during embryogenesis (Figure 3D in revised manuscript). For all earlier stages, we did not see any developmental or size differences. We believe if there were any differences, these would be reflected in size at the mid and late gestational stages we analysed.

- Since our original submission, we have further refined our *in situ* hybridisation protocol on FuChi embryos (Figures 5A and B in revision), finding that strong reporter expression is maintained for all the fluorescent proteins of the H1-Fucci(CA)2 reporter. Therefore, the “notably fainter” appearance of the hGMNN-mVenus in Figure 4A from the first version of the paper was likely a result of the experimental protocol not being 100% optimal.

Minor comments

- We have reordered the paragraphs relating to the different Fucci versions in the introduction as per the suggestions by the reviewer for better clarity.
- To address the issues with Fucci system nomenclatures which made reading difficult, we have now added a background figure (new Figure 1 in revised draft) which is cited in the introduction, made sure constructs are introduced appropriately, and ensured we are consistent with our nomenclature.
- Supplementary Figure lettering corrected.
- All figure panels are now mentioned in the main text, and the incorrect call outs noted by the Reviewer have been corrected
- Removed period and included clarifying statement in the figure legend relating to the comment regarding the extraembryonic region in Figure 5 (original) / Figure 6 (revised).
- Other issues raised relating to reference duplication and missing words have been resolved.
- We have corrected the legend of Figure 1 of the original paper, see related Reviewer 1 response provided above.

**Reviewer #3**

Minor comments

- We have corrected all the figure call outs (see responses to similar comments by Reviewers 1 and 2) to ensure that all data presented is accurately reported.
- In response to Reviewer 3’s comment regarding fluorescent intensity. We quantified fluorescence levels in multiple individual DF1 cells expressing either the H1.0-Fucci(CA)2 or H2B-Fucci(SA)2 reporters, and this is shown as the fluorescent index in Figures 2D, 2E, 2H, and 2I of the revised manuscript, where reporter levels were measured across time. In terms of overall mean intensity levels of the reporters, we found the reporters to be comparable in brightness and have similar mean intensity levels across the cell populations in the flow cytometry data (Figures 2F and 2J).

- We would like to thank the reviewer for suggesting modifications to the cell cycle montages (original figures 1D, 1G and 2F). We agree it would help the reader to enlarge the image, and therefore have reduced the montages to include just one cell cycle, and have also included annotations of cell cycle stages in Figures 2C and 2G of the revised manuscript. We have also added some labels to Figure 3E (original figure 2F) and enlarged this.
- We have added lines to the discussion regarding the use of our reporter in other conventional model systems.

## 4. Description of analyses that authors prefer not to carry out

*Please include a point-by-point response explaining why some of the requested data or additional analyses might not be necessary or cannot be provided within the scope of a revision. This can be due to time or resource limitations or in case of disagreement about the necessity of such additional data given the scope of the study. Please leave empty if not applicable.*

At this stage, we do not decline any of the requests from the reviewers. Where reviewers sought new data or analyses, such as quantification in defined regions of interest, expanded sectioning, improved cross-validation of native reporters with antibody detection, and refined live-imaging presentation, we have either already implemented the necessary changes in the transferred manuscript or outlined concrete plans to do so in the revision. Where concerns stemmed primarily from presentation (e.g., figure labelling, or unclear explanation in figure legends/results), we have addressed many of these already by restructuring figures, enlarging panels, adding explicit stage annotations, and improving legends and textual explanations. Any new data will be presented in a similar way to maintain maximum readability and accessibility.
