# Supplementary material for: FuChi is a cell cycle biosensor for tracking cell cycle dynamics during avian development
Source: PLoS Biol. 2026 Oct 9;24(10):e3004036. doi: 10.1371/journal.pbio.3004036 (PMC13705591; doi:10.1371/journal.pbio.3004036)
Supplement: Supplementary file 3 [file pbio.3004036.s021.docx]

Reviewer #1 (Evidence, reproducibility and clarity (Required)):

SECTION A - Evidence, reproducibility and clarity

Summary:

The manuscript reports the development of a novel Fucci (Fluorescent Ubiquitination-based cell cycle indicator) system for analysing cell cycle analysis, including live imaging of cell cycle. The novel biosensor (H1.0-Fucci(CA)2) has been developed for analyses of chick cells and tissues: chick embryos are a valuable developmental model that have (and in the future, will) particularly informed our understanding of early stages of embryogenesis, and of development of numerous tissues, including the neural tube, somites, limb bud. The authors conclude that the novel system has advantages over previous Fucci systems, including faithful labelling of all four cell cycle phases. Importantly, the authors have generated a stable germline of H1.0-Fucci(CA)2 transgenic chicks, enabling, for the first time, the discrimination and tracking of cells in all 4 phases of the cell cycle - i.e. in vivo studies of cell cycle progression in vivo, in intact tissues and organs. Additional epitope tags mean that the biosensor can be detected in fixed tissues, enabling comparison of cell cycle with expression of mRNA and proteins that mediate other aspects of development/label particular cells and tissues. The authors map proliferation dynamics across numerous tissues in the developing chick, at numerous stages of development, and conclude in particular that transition from S phase may be a key morphogenetic event in gastrulation, as mesendoderm cells leave the primitive streak to form embryonic stuctures such as prechordal plate

Major comments:
The novel biosensor looks to be an incredibly useful tool, and the manuscript suggests patterns of cell cycle progression in different tissues, and at different points in time, that look intriguing. But it is sometimes difficult to draw the strong conclusions suggested by the authors because the text and figures are sometimes difficult to follow. The manuscript would greatly benefit from having someone spend time on the figures, and associated text, to ensure they are fully comprehensible.

*We would like to thank the reviewer for their comments. We have revisited the text, figures, and associated figure legends to ensure that they are more easily accessible and fully comprehensible to readers from across disciplines.*

Specifically:
Conclusion1: That the new FUCCI biosensor is a superior cell cycle probe, better at discriminating all cell cycle phases than previous versions. I was very convinced by the vidoes (video 1 and 2) but had problems with Figure 1. Potentially, this is because I am not an expert in these types of analyses - but it was not helped by the fact that components of the figure were not cited in the text. I was particularly confused by the statement remarking on 'the persistence of mCherry in the H2B Fucci' as mCherry seems to persist longer in H1 (compare Figs 1D and 1G). Please explain, in the Figure legend, why this appears to be the case.

*We apologise for the lack of figure citation in the text. We have now reworked the figures relating to the constructs (original Figures 1 and S1) and have made these Figures 1, 2 and S1 in our updated version. Figure 1 in the revised manuscript is now an introductory background figure which illustrates the differences between Fucci(SA) and Fucci(CA) reporters, with additional details provided in the associated legend, and call outs to the figure starting in the introduction.*

*With regards to statement relating to ‘the persistence of mCherry in the H2B Fucci’ we agree that this could be improved. What we are trying to articulate is that the mCherry degradation that we observed in the Fucci(2A) expressing DF1 cells extended beyond the end of S phase and into G2/M, compared with what would be expected (Revised Figure 2H, arrows). Comparing the hCDT1 reporter in original Figures 1D and 1G, the mCherry persists longer overall in H1.0-Fucci(CA)2 cells vs H2B-Fucci(SA)2 because it accumulates in G1, G2 and M. Additionally, the example shown in Figure 1G of the original submission includes an example of a cell which goes through a very short cell cycle overall. We know by tracking multiple cells, that hCDT1 does persist longer in the H2B-Fucci(SA)2 DF1 cells.*

*We have now replaced these montages with more representative examples. Additionally, the new images (Figures 2C and 2G in the revised manuscript) are synchronised (both starting at G2/M), restricted to a single cell cycle, larger in size, and have the cell cycle stage labelled. We believe these changes will aid interpretation.*

Conclusion 2: that the FuChi chicks are the first viable stably expressing avian cell cycle biosensor model. I agree, and the authors should be congratulated on the development of this important tool.

*We thank the reviewer for their kind words.*

Conclusion 3: the authors monitor cell cycle progression in chicks, in vivo, looking at stages from blastoderm, through gastrulation, and into organogenesis, and draw various conclusions

For example: Fig 3A and text: 'as gastrulation progresses, the primitive streak an presomitic mesoderm display...., whereas the .... And neural plate contains...'

Figure 3A covers an enormous range of stages and tissues. The figure is barely labelled. The text and figure need to better align, and key features in each figure panel need to be labelled so that the reader can better follow, and draw conclusions.

*We agree that Figure 3A in the original submission was not labelled sufficiently, was too expansive, and not enough detail was included in the text or figure legend for readers to follow easily and make their own conclusions. In the revised manuscript, what was Figure 3A is now included as part of a separate and more detailed Figure (Figure 4 in revised manuscript). In this figure, we have added labels to point out anatomical landmarks, and included greater description in the figure legend and text.*

Fig 3B: Reports expression in numerous tissues. There are some beautiful examples of cells segregating relative to cell cycle - for instance, in the neural tube. But I found it hard to know where the biosensor is reporting patterns that are already well established (eg neural tube), and where the biosensor is reporting patterns that are novel - and if so, what these patterns are. Again, this is not described adequately in the text (for instance, there is no mention of the neural tube). And in some cases, references are provided (allowing comparison with previous studies) - but in other cases, there are no references to previous studies. The reader must be given the opportunity to compare this study with previous studies.

*Many of the panels which were originally part of Figure 3B are now included as part of either Figure 4 and 5 in the revised manuscript, alongside new images of different tissues. As above, we have added labels to these images highlighting features of interest to enhance readability. We also now describe in more detail the reporter patterns for all the tissues mentioned including the neural tube (Revised Figures 5A,5B; lines 540-546), the eye (Revised Figure 5C,5D; lines 546-551), brain (Revised Figure 5E; lines 551-553), and intestine (Revised Figures 5G-I; lines 581-603) and discuss these in relation to previous literature highlighting where are findings are novel and/or agreement with previous observations.*

Overall - I can appreciate that there are some fascinating patterns, but it is very difficult to draw the conclusions suggested by the authors. Primarily this is due to poor labelling of figures, and lack of clarity between figures and text, and poor referencing. Additionally, it is not clear that strong conclusions can be drawn about cell cycle in different tissue layers without sectioning some embryos.

*We believe that the inclusion of labels, clearer explanation of the expression patterns, and additional references will help resolve these issues. Additionally, we believe the inclusion of additional images of sections from embryos (Figure 5 in revision), along with quantification of cell cycle status in several tissues (updated Figures 4B, 4G, 5A, 5H and 5I) further support the conclusions we draw from analyses of these embryos.*

Fig 3C: The authors remark 'The results confirm that the ... FuChi embryos recapitulate known cell cycle profiles of those tissues'. See my comments in 3B.

*See response above to comments relating to Figure 3B.*

Conclusion 4: Robust stability of biosensor in fixed tissues. I agree, and the authors should be congratulated for having made a construct that can be paired with in situ hybridisation and immunohistochemistry - this is invaluable.

*We thank the reviewers for their comments.*

Conclusion 5: The authors investigate the potential of the new system for live imaging, and focus on a couple of novel dynamic examples.

The data indicating that PGCs at initial migratory stages are not undergoing frequent cell division is clear.

However, the data indicating that cell cycle status changes as cells egress form the primitive streak, to form prechordal plate, is not clear. The figures need to be better labelled, and the text needs to be more clear (eg ' and prechordal plate. and anterior mesoderm'..

Add labels, pre-chordal plates

*We have added additional labels to Figure 7E and Supplementary Figure S6B in our revision, highlighting the primitive streak and egressing cells of both the overview images and orthogonal sections. We made changes the associated figure legend texts to improve clarity. We have also included a supplementary video showing head fold formation to further demonstrate the cell cycle status of cells that form the pre-chordal plate (also note G1 cells at end of Supplementary video 5 which are egressing anteriorly).*

Minor comments:

-Specific experimental issues that are easily addressable.
I would recommend that the authors section some embryos, to better support key conclusions (eg in figure 3 and 5). *We have now included more tissue sections in revised Figures 4,5, 6 and S5)*
- Are prior studies referenced appropriately?
Not always - see comment above (Fig 3). *We have now added additional appropriate references where are findings match or differ from prior studies.*
- Are the text and figures clear and accurate?
No - this needs work. Not all figures cited in text, or cited in wrong order; Figures are poorly labelled - making it hard to follow. *In our revision we have corrected figure call out issues, and added labels to enhance readability, clarity and data interpretation.*
- Do you have suggestions that would help the authors improve the presentation of their data and conclusions?
Label figures more carefully and ensure figures and text align. *See above.*

**Referee cross-commenting**

I agree with all comments from reviewers 2 and 3

Reviewer #1 (Significance (Required)):

SECTION B - Significance
========================

- Describe the nature and significance of the advance (e.g. conceptual, technical, clinical) for the field.
Technically this is a fantastic resource. As detailed above, the novel biosensor (H1.0-Fucci(CA)2) has been developed for analyses of chick cells and tissues: chick embryos are a valuable developmental model that have (and in the future, will) particularly informed our understanding of early stages of embryogenesis, and of development of numerous tissues, including the neural tube, somites, limb bud. Increasingly, studies show the importance of cell cycle for development, differentiation and morphogenesis - it is a huge breakthrough to be able to perform in vivo studies of cell cycle progression in intact tissues and organs.
- State what audience might be interested in and influenced by the reported findings.
Broad basic research, including developmental biologists, stem cell biologists, modellers.
- Define your field of expertise with a few keywords to help the authors contextualize your point of view. Indicate if there are any parts of the paper that you do not have sufficient expertise to evaluate.
Developmental biologist, with expertise in chick


Reviewer #2 (Evidence, reproducibility and clarity (Required)):

Summary:
This work presents a novel transgenic chicken model with fluorescent reporters that allow in vivo monitoring of the four phases of the cell cycle. To achieve this, the authors clearly identify the limitations of previous Fucci systems and developed an optimised reporter construct that overcomes the major technical challenges identified. Addition of epitope tags to cell cycle stage-specific markers further enables antibody detection in fixed tissues. Proof of concept is provided by live imaging of chick embryos in early developmental stages, evidencing dynamic cell cycle states in tissues and migrating cells.

Major comments:
1. Introduction: Description of the bicistronic tandem-Fucci(CA) system in paragraph 6 is not consistent with what is described in the original bibliographic reference indicated by the authors. Namely: "...accumulation of the CTD1 probe..." should be expected in the G1-S transition (not S-G2) and the yellow reporter should be expected in G2 and M phases (not S and G2, as described). Please review this portion of the text.

*We thank the reviewer for pointing this out. We have now added additional text (lines 79-84) to properly explain the CDT1 probe dynamics, as per the cited manuscript, and also included new schematics (Figure 1 in revision) of the Fucci reporter systems to help readers.*

2. The authors state that "Of note, hatched FuChi chicks are initially smaller than wild type counterparts but grow at comparative rates and are fertile". If the model is to be used to study embryogenesis, it would be useful to indicate if the size of the embryos is comparable to the wildtype, at least for the major developmental stages mentioned in the manuscript.

*We have now included size comparisons of FuChi and wild-type/non-transgenic embryos at mid (E9) and late (E18) gestational stages demonstrating that there is no significant difference between genotypes during embryogenesis (Figure 3D in revised manuscript). For all earlier stages, we did not see any developmental or size differences. We believe if there were any differences, these would be reflected in size at the mid and late gestational stages we analysed.*

3. When referring to somitogenesis, the authors state "...the mesenchyme, which is predominantly in G1 as they undergo condensation". Suppl Video 4, however, shows distinct green fluorescence (S) in the presomitic mesoderm for the first hour or so, only then turning to magenta (G1). The authors should review the sentence/video to clarify if this is a significant finding or if this is not representative of their observations.

*We thank the reviewer for this suggestion. After reviewing our timelapse movies (see Supplementary videos 4 and 6), we agree that presomitic mesoderm does appear to be green (S and/or G2/M phase), which then may transition to G1 as the somites form. To address this further, we analysed cell cycle status in the presomitic mesoderm (Figure 4G in revised manuscript)* *confirming that the majority of cells are in S or G2/M phase. We included a description of these findings in the results (lines 525-530 in revision).*

4. (Optional) It would be interesting to describe if the authors' observations of cell cycle dynamics in the presomitic mesoderm support the proposed Cell Cycle model for somitogenesis (Collier et al., J.Theor.Biol.2000).

*We have included text (lines 776-788 in revised manuscript) discussing our findings in relation to the cell cycle model of somitogenesis.*

5. The fluorescence profiles in Figure 4C do not seem similar regarding the Myc-tag epitope (contrarily to what is stated). The authors should rephrase or revisit this image to clarify their findings.

*We agree that the staining in original Figure 4C were not that similar for the Myc-tag epitope. We have now included a more expansive series of experiments comparing epitope staining on paraffin embedded sections vs cryosections across a range of tissues (Supplementary Figure 5 in revision), confirming the validity of the epitope staining approach.*

6. Quantitative support for several fluorescence-based interpretations made throughout the manuscript. In some instances, conclusions are drawn from qualitative differences in signal intensity. For example, the statement in Fig. 4A that hGMNN-mVenus appears "notably fainter" than the other reporters. Incorporating simple quantitative analyses would strengthen these claims and ensure that observed differences reflect biological behaviour rather than technical or optical factors.
*We thank the reviewer for this suggestion. In our revised manuscript, we have performed quantitative analyses of cell cycle stages in several different tissues and in embryos (Figures 4B, 4G, 5A, 5H, and 5I in revised manuscript). Referring specifically to Figure 4A of the old manuscript, we believe the fainter signal was an experimental artefact. We have further refined our situ hybridisation protocol on FuChi embryos (Figures 6A and 6B in revision), which maintains strong report expression.*

Minor comments:
1. Organization of the information in the Introduction: Paragraphs 3-5 introduce sequentially improved versions of the Fucci system. Then, paragraph 6 returns to the system described in the 4th paragraph. Authors should consider including paragraph 5 (description of Fucci4 and its limitations) just prior to the description of chickens as valuable developmental models (current paragraph 8) for clarity of the text.

*We have reordered these in the text.*
2. Fucci system nomenclature. Many different Fucci systems are mentioned, but nomenclature consistency throughout the manuscript is lacking, which makes reading difficult. For example, the terms "Fucci(SA)2" and "Fucci(CA)2" should be defined in the introduction, as they are employed to describe the construction of the new biosensor in the following sections.

*Thank you for this suggestion. We have now added a background figure (new Figure 1 in revised draft) that is cited in the introduction, and have made sure we are consistent on our nomenclature.*
3. Some figure panels are not mentioned in the main text (for ex. Figures 1B and C, Figure 2C). *We have now addressed this.*
4. The legend of Figure 1 (D & G) mentions "denoted by *", but the * seems to be missing in the figure.

*We have now replaced this figure. See response to Reviewer 1.*
5. Supplementary Figure 1 has two D panels (and is missing the E).

*This has been corrected.*
6. In the main text, where it reads "...Flow cytometry analysis of three independent PGC lines... (Figures 2G & S2E)", S2E should be replaced by S1E.

*This has been amended to the correct figure call out.*
7. In the Figure 4A legend, hCDT1-mVenus should be corrected to hCDT1-mcherry. Also, it is not clear why the authors state that "hGMNN-mVenus expression is notably fainter compared with hCDT1-mVenus and H1.0-mCerulean expression".

*This label has now been corrected; see response above regarding fainter GMNN-Venus expression.*
8. In Figure 5E, the optical sections "i" seem to pertain to the extraembryonic tissue/area opaca and not to anterior mesoderm, as stated in the figure legend. Also, there is a period between "prechordal plate" and "and" in the legend's last sentence.

*Removed period and included clarifying statement in the figure legend.*
9. Discussion: The last sentence of the third paragraph lacks "to" between "used" and "interrogate".

*Corrected*
10. References 10 and 23 are identical.
*Corrected*
**Referee cross-commenting**

I agree with all comments from reviewers 1 and 3

Reviewer #2 (Significance (Required)):

This is a beautiful paper, describing a long sought-after model system to study cell cycle dynamics in vivo. The methodological details are thorough, and the results obtained are clearly presented, highlighting the utility of the new model in various embryonic stages and tissues/organs.

This work is of pivotal importance to the developmental/stem cell biology community, as well as to the wider community that employs the chicken embryo as a preclinical model to assess therapeutic or teratogenic potential of biologically- or chemically-derived products.

*We thank the reviewer for their kind words and suggestions.*

My expertise is in chicken embryo development, namely gastrulation, somitogenesis and limb bud outgrowth.


Reviewer #3 (Evidence, reproducibility and clarity (Required)):

The manuscript by Sudderick and colleagues describes the development and characterisation of a new generation of cell cycle reporter that can distinguish between cells in G1, S, G2 and M phases. Furthermore, the authors have developed a transgenic chicken line incorporating this reporter and demonstrated faithful discrimination of cell cycle stages in the in vivo context of developing transgenic embryos. Of note is the addition of epitope tags, which facilitate discrimination of cell cycle stages in tissue fixed using various techniques. This is a very important paper for the following reasons:

• The authors have achieved faithful discrimination of all four cell cycle stages, which is a major advance in itself.
• This generation of the FuChi transgenic chick is of enormous importance. This will facilitate accurate in vivo studies in a broad range of fixed and living tissue types and is a major milestone in the further establishment of the chick as a transgenic model system.

Th characterisation of the cell cycle reporter as presented is robust and convincing. The authors further demonstrate the potential utility of the FuChi chickens through their observation of partial cell cycle synchrony during onset of development. I therefore only have minor suggestions that may facilitate easier interpretation of their data.

Results 2

• I can't see any mention of Figures 1C and D. Presumably the authors have carried out fluorescence intensity measurements using the two cell cycle reporters here, but this is not mentioned in the main text.

*We have now corrected the figure call outs (see responses to similar comments by Reviewers 1 and 2) to ensure that all data presented is accurately reported.*

• Figure 1D&G: I find these difficult to follow given the small size of the cells as presented. The authors may consider enlarging these and clearly annotating for cell cycle stage. They may find it helpful to focus on a single cell cycle, although I appreciate that displaying two cell cycles strengthens the claim of efficacy of the newly developed sensor. The supplementary videos associated with these figure panels are excellent as they display several cells with faithful reporter activity, but again, the authors may wish to annotate a few of these cells to enhance speedy interpretation. I have similar comments for Figure 2F and the associated movie.

*We thank the reviewer for this suggestion. We agree it would help the reader to enlarge the image and we have therefore reduced the montage to include 1 cell cycle, and have also included annotation of cell cycle stages (Figures 2C and 2G in revised manuscript). We have added some labels to Figure 3E (original figure 2F) and enlarged this. We have also processed the supplementary videos to include annotations highlighting dividing cells to help readers interpret the data more readily.*

Results 4

• The authors state that a large proportion of blastoderm cells were in G2/M. They may wish to formally quantify this, perhaps by performing simple cell counts in designated regions of interest. A similar quantification for gastrulating embryos would also be helpful.

*See comments to Reviewer 2. We have now quantified cell cycle status in embryos and several tissues, including blastodermal cells (Figure 4B in revision) as suggested. This confirmed the large presence of G2/M nuclei in the blastoderm.*

• It would be helpful to see zoomed in images of selected regions of the somites, neural tube and retina displayed in Figure 3B. This would be particularly appropriate in the context of the neural tube and retina (which are not discussed in the main text) as the positioning of the nucleus is defined by the stage of the cell cycle and should therefore serve to highlight the efficacy of the reporter.

*We thank the reviewer for this suggestion. We have now included higher magnification of dividing cells in the neural tube (new Figure 5B) and retina (new Figure 5D). We found many examples of hGMNN positive cells (which is cytoplasmic at M phase) undergoing cell division in the perpendicular plane, as previously reported for both tissues (Das et al., 2012; Silva et al, 2002). Descriptions of these events are now included in the text (lines 542-544; lines 549-551) and placed in context with previous literature.*

• Video 4 beautifully demonstrates the high levels of proliferation in somites, but again, it would be useful to have a zoomed in view. I appreciate the difficulty involved in doing this, given the movement of the embryo, but perhaps the authors could focus on a fixed ROI or present a separate movie of a few cells undergoing a full cell cycle.

*We have included videos with a fixed ROI focused on an individual somite, enabling proliferation in individual cells of the forming somites to be more readily visualised (second part of Supplementary video 6 in revision).*

Discussion

• The authors could perhaps expand on their discussion about potential utility in other conventional model systems (e.g. mouse, fish, etc).
*We have included additional examples of suggested uses for our reporter construct in other systems (lines 794-798)*


Reviewer #3 (Significance (Required)):

General assessment: A timely piece of work that introduces a faithful cell cycle reporter that will be of broad interest.

Advance: The ability to discriminate between all four stages of the cell cycle is a clear advance here.

Audience: Broad interest, including those studying cell cycle and embryonic development in several tissue contexts.

Expertise: Chick embryology, in vivo live imaging, neurogenesis, cellular developmental biology

*We would like to thank the reviewer for their suggestions and kind words regarding our manuscript.*
