# Supplementary material for: FuChi is a cell cycle biosensor for tracking cell cycle dynamics during avian development
Source: PLoS Biol. 2026 Oct 9;24(10):e3004036. doi: 10.1371/journal.pbio.3004036 (PMC13705591; doi:10.1371/journal.pbio.3004036)
Supplement: Supplementary file 4 [file pbio.3004036.s022.docx]

Response to Reviewer

Reviewer #2: Sudderick et al present a beautiful work describing a new in vivo model of cell cycle progression with widespread applications. They evidence its flexibility and applicability to the study of embryo development, providing new intriguing results and future avenues. This work builds on a previously reviewed manuscript, having largely answered to the concerns appointed by the reviewers. I strongly support its publication with minor alterations

*We would like to thank the reviewer for their kind words and for the time and effort they have taken to review our manuscript.*

Minor comments:

-	Author affiliation numbers 6 and 7 should be listed in order of appearance.

*We have changed the author affiliation numbers and list so that they match the order in which they appear.*

-	Line 110: "Geminin" is used without having previously been defined as GMNN.

We have defined both Geminin (GMNN) and Chromatin licensing and DNA replication factor 1 (CDT1) in line 63 of the revised manuscript.

-	Line 385: "Figure S1B" should read "Figure S1B,C".

*We have corrected this figure callout. This now appears in line 406 of the revised manuscript.*

-	Figure 1C, D appear to contain the same information as Figure 2A, although "Fucci(SA)" and "Fucci(CA)" are used in Fig1 and "Fucci(SA)2" and "Fucci(CA)2" in Fig2. It is also not clear if image repetition is relevant.

*While these panels are similar in appearance, there are distinct and relevant differences. Figures 1C and 1D show the generic Fucci(CA) and Fucci(SA) systems, and therefore specific fluorescent proteins are not labelled. In contrast, Figure 2A shows the specific version 2.0 Fucci(CA) and Fucci(SA) biosensors used in this study, which contain mCherry and mVenus. We have used this terminology to remain consistent with the established nomenclature for these reporter systems.*

-	Figure 3D shows results of embryo weight (g). However, this is referred to as "size" in the figure title, legend and manuscript text (e.g. line 433 uses the term "smaller"). Since these embryos may be a relevant tool for tissue elongation studies (size measurements in mm), it should be clarified in the text that "weight" is being used as an overall estimate of organism size in this work.

*We have now added the following qualifying statement to clarify that weight is being used as an overall estimate of size (lines 451-455 in revision):*

*“…..comparable to non-transgenic embryos by visual assessment and by weight as an overall estimate of size (Fig 3D)”.*

-	Line 473: "Figure S4" should read "Figure S3". Also, Figure S3 and Figure S4 in the PDF file do not match the TIFF files nomenclature.

*Thank you for noticing this. We have now corrected the figure call out, and file uploads.*

-	Line 541: The phrasing seems misleading, since apical cells are also predominantly in G1 (~50%), although this is significantly increased in basal cells. Please rephrase for clarity.

*We have rephrased the aforementioned text (lines 563-568 in revised manuscript) to avoid any chance of misinterpretation of the data and also to improve clarity.*
